# Epstein–Barr virus nuclear antigen 3A protein regulates CDKN2B transcription via interaction with MIZ-1

**DOI:** 10.1093/nar/gku697

**Published:** 2014-08-04

**Authors:** Quentin Bazot, Thibaut Deschamps, Lionel Tafforeau, Maha Siouda, Pascal Leblanc, Marie L. Harth-Hertle, Chantal Rabourdin-Combe, Vincent Lotteau, Bettina Kempkes, Massimo Tommasino, Henri Gruffat, Evelyne Manet

**Affiliations:** 1 CIRI, International Center for Infectiology Research, Oncogenic Herpesviruses team, Université de Lyon, Lyon 69364, France; 2 Université Lyon 1, Centre International de Recherche en Infectiologie, Lyon 69364, France; 3 CIRI, International Center for Infectiology Research, Cell Biology of Viral Infections team, Université de Lyon, Lyon 69364, France; 4 INSERM, U1111, Lyon 69364, France; 5 CNRS, UMR5308, Lyon 69364, France; 6 Ecole Normale Supérieure de Lyon, Lyon 69364, France; 7 International Agency for Research on Cancer, World Health Organization, Lyon 69372, France; 8 CNRS UMR5239, Laboratoire de Biologie de la Cellule, Lyon 69364, France; 9 Department of Gene Vectors, Helmholtz Center Munich, German Research Center for Environmental Health, Munich, Germany

## Abstract

The Epstein–Barr virus (EBV) nuclear antigen 3 family of protein is critical for the EBV-induced primary B-cell growth transformation process. Using a yeast two-hybrid screen we identified 22 novel cellular partners of the EBNA3s. Most importantly, among the newly identified partners, five are known to play direct and important roles in transcriptional regulation. Of these, the Myc-interacting zinc finger protein-1 (MIZ-1) is a transcription factor initially characterized as a binding partner of MYC. MIZ-1 activates the transcription of a number of target genes including the cell cycle inhibitor *CDKN2B*. Focusing on the EBNA3A/MIZ-1 interaction we demonstrate that binding occurs in EBV-infected cells expressing both proteins at endogenous physiological levels and that in the presence of EBNA3A, a significant fraction of MIZ-1 translocates from the cytoplasm to the nucleus. Moreover, we show that a trimeric complex composed of a MIZ-1 recognition DNA element, MIZ-1 and EBNA3A can be formed, and that interaction of MIZ-1 with nucleophosmin (NPM), one of its coactivator, is prevented by EBNA3A. Finally, we show that, in the presence of EBNA3A, expression of the MIZ-1 target gene, *CDKN2B*, is downregulated and repressive H3K27 marks are established on its promoter region suggesting that EBNA3A directly counteracts the growth inhibitory action of MIZ-1.

## INTRODUCTION

Epstein–Barr virus (EBV) is a ubiquitous herpesvirus associated with several human cancers, both in immuno-competent individuals (Burkitt's lymphoma, Hodgkin's disease (HD), rare T-cell and NK-cell lymphomas, undifferentiated nasopharyngeal carcinoma (NPC), gastric carcinomas) and immuno-compromised individuals (lymphoproliferations and lymphomas, in particular in post-transplant patients) (1). The primary infection is usually asymptomatic when it occurs in childhood but later in life can result in the infectious mononucleosis (IM). Following the primary infection, the virus persists in a life-long latent state in the memory B cells of infected individuals with intermittent viral production occurring in the oropharynx. *In vitro*, EBV has the unique capacity to induce growth transformation of resting primary human B-lymphocytes upon their infection, leading to the establishment of lymphoblastoid cell lines (LCLs). In such cell lines, there is no virus production but nine viral proteins—six nuclear antigens (EBNAs 1, 2, 3A, 3B, 3C and LP) and three membrane proteins (LMPs 1, 2A and 2B)—as well as several non-coding RNAs and miRNAs are expressed, that act in concert to induce and maintain continuous proliferation (for a review see (2,3)).

The EBNAs 3A, 3B and 3C genes are arranged in tandem in the EBV genome and are thought to originate from a series of gene duplication events. Their structure is very similar, with a short 5′-coding exon and a long 3′-coding exon, and the resulting proteins share limited but significant amino acid sequence homology, especially in their N-terminal (NT) domains. Initial genetic studies using EBV recombinants suggested that EBNA3A and EBNA3C (but not EBNA3B) are absolutely required for the immortalization of B lymphocytes *in vitro* (4). Moreover, LCLs that express conditional EBNA3A or EBNA3C mutant proteins, cease to proliferate in the absence of functional EBNA3A or EBNA3C respectively (5,6). Recently, however, Hertle *et al.* succeeded in establishing LCLs infected with a recombinant EBV deficient for EBNA3A, although these cell lines exhibit reduced proliferation rates and elevated levels of apoptosis (7).

The EBNA3s are functionally pleiotropic proteins that have the properties of transcription factors but which are also involved in the control of cell proliferation by directly interacting with proteins such as p53 (8) or Cyclin A (9) and in the regulation of proteasome-dependent protein degradation by interacting with protein complexes such as SCF^SKP2^ (10,11). EBNA3A and EBNA3C can cooperate with H-RAS in the immortalization and transformation of rat embryonic fibroblasts (REFs) and relieve suppression of REF transformation by the Cyclin D-dependent kinase inhibitor (CDKI) p16^INK4A^ (12–14). Through their capacity to regulate transcription, the EBNA3s appear to have important cell cycle-associated activities as well as anti-apoptotic functions. In particular, EBNA3A and EBNA3C cooperate to repress the expression of the pro-apoptotic tumor-suppressor gene *BCL2L11* (*BIM*) (15) and the cyclin-dependent kinase inhibitor gene *CDKN2A* encoding p16^INK4a^ (16,17).

The role of the EBNA3s as transcriptional regulators was first characterized by the use of heterologous Gal4-dependent reporter gene assays because they do not interact with DNA directly. As full-length proteins, EBNA3A and EBNA3C appear to act as transcriptional repressors, but various domains of both proteins have been reported to exhibit either an activating or repressive activity (18–22). Transcriptional activation by the EBNA3 proteins could be linked to their interaction with prothymosin alpha (PTMA) and the histone acetyltransferase (HAT) p300 (23,24). Repression by the EBNA3s has been shown to be mediated by various co-repressor proteins or protein complexes including C-terminal (CT)-binding protein 1 (CTBP1) (13,14) and the histone deacetylases, HDAC1 and 2 (25,26). Furthermore, it was recently shown that, by acting together, EBNA3A and EBNA3C can trigger the recruitment of polycomb repressive complex 2 (PRC2) core subunits to the *BIM* promoter, leading to local trimethylation of histone H3 on lysine 27 (H3K27me3) (27).

More recently, transcriptomic studies have revealed the extent of the impact of the EBNA3s on cellular transcription, with over 1000 host cell genes found to be regulated by one or different combinations of the EBNA3s in B cells (7,28–32). In addition, ChIP-sequencing (ChIP-seq) analyses have identified up to 13 000 genomic sites for the EBNA3s (32,33). However, cellular factors that recruit the EBNA3s onto the chromatin have not as yet been well defined except for the cellular DNA-binding factor RBPJ (also called RBP-Jk or CBF1). RBPJ also binds and targets both the EBV transcriptional activator EBNA2 and the Notch-IC effector of the Notch signaling pathway to DNA. Acting alone, RBPJ is a transcriptional repressor that recruits co-repressor complexes to its target promoters. By interacting with RBPJ bound to specific DNA sequences in the EBV C- and LMP1/LMP2 promoters, EBNA2 appears to displace the RBPJ co-repressor complex and thus activates the transcription of most EBV latency genes—including the EBNA3s—expressed in the LCLs. In transient reporter gene assays, the EBNA3s have been shown to interfere with this RBPJ-dependent activation of the viral C- and LMP1/LMP2A promoters by EBNA2, and are thus believed to be part of an auto-regulatory feedback loop controlling EBNA2 as well as their own expression (18,20,22,34,35).

In order to better understand the mechanisms involved in the multiple functions of the EBNA3s, we used a large-scale yeast two-hybrid (Y2H) screen to identify the principal interactions which each of the EBNA3 proteins makes with the cellular proteome. From this screen, we have identified a number of relevant interactions between the EBNA3s and cellular proteins, some of which allow the formulation of new hypotheses that potentially fill the gaps in our knowledge regarding the mechanisms of action of these proteins. In particular, among the new interactors we have identified, are several transcriptional regulators that bind the core promoter elements of a number of genes known to govern cell cycle progression. We focused our attention on one of them, ZBTB17, also known as MYC interacting finger protein 1 (MIZ-1). MIZ-1 is a BR-C, ttk and Bab (BTB)/Pox virus and Zing finger (POZ) domain-containing transcription factor that binds to the transcription initiator site (Inr) in the core promoters of target genes (36,37) and depending on its binding partners, can function as an activator—by recruiting co-activators such as p300 or the multifunctional cellular chaperone protein, nucleophosmin (NPM, also known as B23, NO38 and Nutramin) (38)—or as a repressor. One of the important partners of MIZ-1 is MYC, which by binding MIZ-1, can replace the activating co-factors of MIZ-1. In the absence of MYC, MIZ-1 activates expression of its target genes in response to anti-mitogenic signals such as treatment with growth inhibitory cytokine growth factor-β (TGFβ) (37,39) and exposure to DNA damage (40). Thus, whereas MIZ-1 alone has growth arrest activity by activating the expression of target genes such as *CDKN1A* (encoding p21^WAF1^), *CDKN2B* (encoding p15^INK4b^) and *CDKN1C* (encoding p57^KIP2^), the MAX–MYC–MIZ-1 complex represses their expression (for a review see (41)). Other transcriptional regulators such as BCL-6 (42,43), GFI-1 (44) and HCF-1 (45) have been shown to interact with MIZ-1 and repress the transcription in a similar way to MYC. Furthermore, the p14ARF tumor suppressor protein has been shown to both disrupt the interaction between MIZ-1 and NPM, and induce MIZ-1 sumoylation (46).

We show here that all three EBNA3 proteins interact with MIZ-1. Focusing on EBNA3A we show that its expression in LCLs is responsible for transcriptional repression of the MIZ-1-specific target gene *CDKN2B*. Furthermore, we show that EBNA3A expression induces delocalization of MIZ-1 from the cytoplasm to the nucleus and correlates with an increase in the H3K27me3 mark at the *CDKN2B* promoter. Finally, we provide evidence that EBNA3A also represses MIZ-1 target gene expression by interfering with the recruitment of its coactivator, NPM.

## MATERIALS AND METHODS

### Plasmids

Open Reading frames (ORFs) for full-length EBNA3A, EBNA3B, EBNA3C, EBNA3A-Nter (aa 1 to 310), EBNA3B-Nter (aa 1 to 325), EBNA3C-Nter (aa 1 to 320), EBNA3A-Cter (aa 274 to 944), EBNA3B-Cter (aa 279 to 938) and EBNA3C-Cter (aa 284 to 992) were cloned using the Gateway recombinational cloning system (47) and deposited in a viral ORF repository, viralORFeome (48). Each ORF was polymerase chain reaction (PCR)-amplified (KOD polymerase, Novagen) from pSG5-EBNA3A/3B/3C (20) using reverse and forward primers containing the *attB1* and *attB2* recombination sites respectively, then cloned into pDONR207 (BP Clonase, Invitrogen). The ORFs were subsequently transferred (LR Clonase, Invitrogen) into the bait vector, pGBKT7, to be expressed as GAL4-DB fusions in yeast. Expression plasmids for EBNA3A, 3C, 3B tagged with the Flag or Myc epitopes were obtained by recombinational cloning from the pDONR207 vectors into expression vectors pCI-neo-3xFlag-gw and pDEST-myc (kindly provided by Dr Y. Jacob) respectively. Expression plasmids for the cellular proteins identified in the Y2H screen were obtained either by recombinational cloning using a human ORFeome library (cloned into pDONR223) into expression vector pDEST-myc or by RT-PCR amplification from HeLa cells mRNA or were kindly given by Dr J. Lukas (FBXO18) (49), Dr M. Eilers (MIZ-1) (37), Dr K. Tang (CENPJ) (50), Dr A. M. Naar (MED25) (51), Dr E. Krueger (PSMA7) (52). MIZ-1 and EBNA3A deletion mutants were generated by cloning the corresponding PCR-amplified fragments into pDONR207, which were transferred into pCI-neo-3xFlag-gw or pDEST-myc. EBNA3A deletion mutant Δ141–238 and EBNA3A-CtBPmut, in which the two amino acid motifs, ALDLS (aa 857–861) and VLDLS (aa 886–890) are substituted by ALDAA and VLDAA respectively, were generated by site-directed mutagenesis (QuickChange Site-Directed Mutagenesis kit, Stratagene). The *CDKN2B* (−113- +160) Luc reporter construct was a kind gift from Dr Eilers. The CMV-Renilla Luciferase plasmid used as an internal control in the transfection has been described previously (53).

### Yeast two-hybrid screens

The screens were made using matings between the AH109 and Y187 yeast strains (Clontech) (54,55). Bait vectors, pGBKT7-EBNA3A/3A-Nter/3A-Cter, pGBKT7-EBNA3B/3B-Nter/3B-Cter, pGBKT7-EBNA3C/3C-Nter/3C-Cter were transformed into AH109 (bait strain). A human LCL AD-cDNA library (56) and a human spleen normalized AD-cDNA library (Invitrogen) were transformed into Y187 (prey strain). Single bait strains were mated with prey strain, then diploids were plated on SD-W-L-H + 5 mM 3-AT medium. Positive clones were maintained on this selective medium for 15-days to eliminate any contaminating AD-cDNA plasmid (57). AD-cDNAs were amplified by colony PCR and inserts were sequenced and identified by automatic Basic Local Alignment search Tool (BLAST) as described previously (58).

### Cell culture and transfections

HeLa cells and HEK293T cells were grown at 37°C in Dulbecco's modified Eagle's medium supplemented with 10% fetal bovine serum (FBS) and penicillin-streptomycin. LCLs generated from B cells derived from two different donors—donor 1 (D1) and donor 2 (D2)—and infected with either wild-type (wt) recombinant EBV (D1wt, D2wt) or EBNA3A knock-out virus (D1E3AmtA, D2E3AmtB1, D2E3AmtB2, D2E3AmtB3) have been described previously (7) as well as the ΔE3A-^LCLdoxE3A^ LCL (31) and were maintained in Advanced RPMI 1640 medium supplemented with 10% FBS and penicillin-streptomycin. Plasmid transfection was performed using either the calcium phosphate precipitate method or the PEI transfection reagent (Polysciences). Treatment of LCLs with the MYC inhibitor, 10058-F4 (Calbiochem) was performed at a concentration of 64 μM for 24 h. Treatment with cisplatin (Sigma-Aldrich) was performed at 33 μM concentration.

### Flow cytometry analysis

For quantification of NGFR-expressing cells, cells were stained with APC-coupled mouse anti-human CD71 (NGFR) antibody (BioLegend). For cell cycle analysis cells were stained with Propidium Iodide (Abcam) using standard protocols. Fluorescence of cells was detected and analyzed using a FACSCalibur system and CellQuest Pro software (BD Biosciences).

### Co-immunoprecipitation assays and western blots

Transfected HEK293T or HeLa cells were harvested from 100 mm dishes 48 h post-transfection and lysed in IP buffer (50 mM Tris-HCl pH 7.5, 150–300 mM NaCl, 1mM Dithiotreitol (DTT) and 0.5% Nonidet P-40) plus protease inhibitors (Roche Molecular Biochemicals). For immunoprecipitation of the transiently expressed Flag-tagged proteins, cell extracts were incubated with 30 μl anti-Flag M2 affinity gel (Sigma) for 4 h at 4°C and the immunopurified proteins were analyzed by western blotting. Antibodies used for other immunoprecipitations or western blotting analysis were anti-Flag rabbit polyclonal antibody (Sigma), anti-Myc (9E10) mAb, anti-human MIZ-1 goat polyclonal antibody (R & D Systems), anti-EBNA3A sheep polyclonal antibody (Exalpha biologicals Inc), anti-Nucleophosmin mAb (Invitrogen) and anti-α-tubulin mAb (B-5–1–2, Sigma). The appropriate anti-mouse (GE Healthcare), anti-rabbit (GE Healthcare), anti-goat (Santa Cruz Biotechnology, Inc) or anti-sheep (Abcam) horseradish peroxidase (HRP)-conjugated antibodies were used as secondary antibodies. Myc-tagged proteins were also revealed using Myc (9E10) HRP-conjugated antibody (Santa Cruz Biotechnology, Inc). Western blots were revealed using Enhanced Chemiluminescence (ECL) (Pierce).

### *In vitro* GST Pull-down assays

Glutathione S-Transferase (GST) and GST-fusion proteins were purified from *Escherichia coli* BL21 (DE3) codon plus strain extracts, with glutathione-Sepharose 4B beads (GE Healthcare). Beads carrying the GST or the GST-fusion proteins were equilibrated in the following binding buffer (10 mM Tris-HCL pH 8, 250 mM NaCl, 0.1% NP40 and 2 mg/ml bovine serum albumin (BSA)) in the presence of protease inhibitors (complete EDTA-free cocktail from Roche Molecular Biochemicals) and incubated with radiolabeled proteins synthesized *in vitro* in the presence of [^35^S]-methionine using the TnT Coupled Transcription/Translation system (Promega), in binding buffer for 4 h at 4°C. Beads were washed 5× in binding buffer without BSA and bound proteins were fractionated by sodium dodecylsulphate-polyacrylamide gel electrophoresis (SDS-PAGE) and revealed by autoradiography.

### Oligo pull-down assays

Cells were lysed by sonication in Hepes, KCl, MgCl2, Glycerol (HKMG) buffer (10 mM HEPES, pH 7.9, 100 mM KCl, 5 mM MgCl2, 10% glycerol, 1 mM DTT, 0.5% NP40) containing protease and phosphatase inhibitors. Cellular debris was removed by centrifugation. Then, 1 mg of total lysate was precleared with 40 μl of streptavidin-agarose beads (Thermo Scientific) for 1 h at 4°C, with rotation, and incubated with 2 μg of biotinylated double-stranded oligonucleotide (Table S1, Supplementary Data) and 20 μg of poly(dI-dC) for 16 h at 4°C, with rotation. Biotin-oligonucleotide-protein complexes were collected with 60 μl of streptavidin-agarose beads for 1 h at 4°C with rotation, washed twice with HKMG buffer, separated on SDS-PAGE, and detected by western blotting.

### RNA analysis

RNAs were prepared using the Nucleospin RNA kit (Macherey-Nagel) and reverse transcribed using the qScript™ cDNA SuperMix (Quanti Biosciences). Standard PCRs were performed using the GoTaq^®^DNA polymerase (Promega) and the PCR-amplified fragments were analyzed on 2% agarose gels. We used ß-actin mRNA as internal control. Amplification of a single 690-bp DNA fragment corresponding to the ß-actin mature mRNA showed that no DNA contamination was present in our RNA preparations. qPCR was performed using FastStart Universal SYBR Green Master (Rox) (Roche Molecular Biochemicals) on an Applied Biosystems 7000 thermocycler. Cycling conditions were 5 min at 95°C and 45 cycles of 15 s at 95°C, 30 s at 60°C on a 96-well thermoblock. This program was followed by melting curve analysis in order to verify the specificity of the PCR product. PCR results were normalized with the parallel amplification of *GAPDH* mRNA. The various set of primers used in the study are listed in Table S1 of Supplementary Data.

### Immunofluorescence assays

For indirect immunofluorescence experiments, anti-Flag polyclonal antibody (Sigma) and anti-Myc 9E10 mAb were used as primary antibodies. Alexa Fluor 488 goat anti-rabbit antibody (Invitrogen) and Fluorolink Cy3-labeled goat anti-mouse IgG (H + L) (GE Healthcare) were used as secondary antibodies. Cell nuclei were stained by incubation with 0.5 μg/ml Hoescht 33342 (Sigma). Slides were examined using confocal microscopy (Zeiss LSM 710) with ZEN software for the photos and standard microscopy (Zeiss Axiovert 135) with Image J software for counting the cells.

### Luciferase Assays

Renilla or Firefly luciferase activities from transfected cells were measured in a Veritas™ Luminometer (Turner Biosystems) using the Renilla or Firefly Luciferase Assay system (Promega Madison Co). Luciferase activity was measured for identical amounts of total protein as evaluated by Bradford assay.

### Chromatin immunoprecipitations

Chromatin immunoprecipitations (ChIPs) were performed as described in the Upstate protocol. Briefly, lymphoblastoid cells were cross-linked with 1% formaldehyde at RT for 10 min. Crosslinked chromatin was sonicated using the Bioruptor^®^ from Diagenode in order to obtain sheared chromatin, yielding fragments of 150 to 500 bp. Sheared chromatin generated from the equivalent of 1–2 × 10^6^ cells was used per ChIP. Antibodies used to immunoprecipitate methylated histones were rabbit polyclonal anti-trimethyl-Histone H3 (Lys27) antibody (07–449 Millipore) and anti-dimethyl-Histone H3 (Lys4) mAB (clone AW30; 04–790 Millipore). Rabbit IgG (PP64 Millipore) or mouse IgG (PP54 Millipore) were used respectively as negative control antibodies. Precipitated DNA was assayed by quantitative PCR as described in the RNA analysis section and the amount of immunoprecipitated chromatin was expressed from duplicate assays as percentage of input chromatin. The different primers used are listed in Table S1 of Supplementary Data.

## RESULTS

### Identification of novel cellular interacting partners for EBNA3A, EBNA3B and EBNA3C by yeast two-hybrid screening

We used the Y2H system to identify cellular proteins that interact with the EBNA3 family of proteins. Six constructs encoding either full-length, NT or CT regions of EBNA3A, EBNA3B and EBNA3C were used to probe both a human LCL cDNA library and a human spleen cDNA library. Five hundred ninety-seven cDNA clones were initially characterized following PCR amplification of the cDNAs and sequencing. From these, a majority were identified as corresponding to RBP-J cDNAs. The characterization of the rest of the clones allowed us to identify 35 different putative interactors. We then validated the interactions between the putative novel cellular partners and each of the EBNA3 proteins via co-immunoprecipitation assays in HEK293T cells, following transfection of expression plasmids encoding full-length proteins. Twenty-seven cellular proteins were thus identified as true cellular EBNA3-interacting proteins (Table 1 and Supplementary Figure S1). Most appeared to interact with all three EBNA3 proteins, even though they were often identified only with one EBNA3 in the Y2H screen. However, it is to be noted that in several cases we observed important differences in the efficiency of co-immunoprecipitation of a specific partner depending on the EBNA3 used.

**Table 1.** List of cellular proteins interacting with EBNA-3A/-3B/-3C identified in the Y2H screens and validated *in cellulo* by co-immunoprecipitations in HEK293T cells

GeneID	Hits	Gene symbol	Full name	Y2H	CoIP
3516	475	RBPJ*	Recombination signal binding protein for immunoglobulin kappa J	A B C	ND
1487	25	CTBP1*	C-terminal binding protein 1	A C C_Ct_	ND
7791	11	ZYX*	Zyxin	B B_Ct_	A B
55835	6	CENPJ*	Centromere protein J	B B_Ct_ C	A C
3337	6	DNAJB1*	DnaJ (Hsp40) homolog, subfamily B, member 1	A	A B C
7709	6	*ZBTB17*	Zinc finger and BTB domain containing 17	B C_Ct_	A B C
		*MIZ-1*^#^	*Myc-Interacting Zn Finger Protein 1*^#^		
84893	5	*FBXO18*	F-box protein, helicase, 18	C C_Nt_	A C
151887	4	*CCDC80*	Coiled-coil domain containing 80	C C_Ct_	A B C
10987	4	*COPS5*	COP9 constitutive photomorphogenic homolog, subunit 5	A_Nt_ C_Nt_	A B C
23557	3	*SNAPIN*	SNAP-associated protein	A_Nt_ C_Nt_	A B C
6613	3	*SUMO2*	SMT3 suppressor of mif two 3 homolog 2	C_Ct_	A B C
1488	2	*CTBP2*	C-terminal binding protein 2	A	A B C
4802	2	*NFYC*	Nuclear transcription factor Y, gamma	A	A B
5536	2	*PPP5C*	Protein phosphatase 5, catalytic subunit	A	A B C
409	1	*ARRB2*	Arrestin, beta 2	A	A B C
8665	1	EIF3F*	Eukaryotic translation initiation factor 3 subunit F	C	A B C
3190	1	*HNRNPK*	Heterogeneous nuclear ribonucleoprotein K	C_Nt_	C
3843	1	*IPO5*	Importin 5	A_Nt_	A B C
10445	1	*MCRS1*	Microspherule protein 1	C_Ct_	A B C
81857	1	*MED25*	Mediator complex subunit 25	A	A B C
4222	1	*MEOX1*	Mesenchyme homeobox 1	A	A B C
9260	1	*PDLIM7*	PDZ and LIM domain 7	C_Ct_	A B C
5688	1	*PSMA7*	Proteasome subunit, alpha, type 7	C_Nt_	A B
6136	1	*RPL12*	Ribosomal protein L12	C	A B C
6138	1	*RPL15*	Ribosomal protein L15	A	A B C
10963	1	*STIP1*	Stress induced phosphoprotein 1	A	A B C
79090	1	*TRAPPC6A*	Trafficking protein particle complex 6A	A	A B C

Each human gene is identified by its National Center for Biotechnology Information (NCBI) gene ID (first column), gene symbol (third column) and full name (fourth column). The number of hits indicated (second column) is given without discrimination of the bait protein. Column 5 indicates with which bait protein(s) the cellular partner has been identified in Y2H. Column 6 indicates which of the EBNA-3A/-3B/-3C have been validated with the cellular partner by co-immunoprecipitation *in cellulo*. A: EBNA3A, B: EBNA3B, C: EBNA3C, A_Nt_ : EBNA3A (1–310), B_Ct_ : EBNA3B (1–325), C_Nt_ : EBNA3C (1–320), C_Ct_ : EBNA3C (279–938). ND: Not Done. * Indicates cellular partners for which an interaction with at least one of the EBNA3 was previously described. ^#^ Alternative name.

From our list of cellular interactors, six (namely RBPJ (20,59–61), CTBP1 (13,14,17), ZYX (62), CENPJ and EIF3F (63)) had been previously described as direct interactors of at least one of the EBNA3 proteins. Interestingly, our systematic validation, by co-immunoprecipitation in a mammalian expression system, of interaction with the three EBNA3s allowed us to complete the description of these previously known interactions. ZYXIN was previously identified in a Y2H screen using EBNA3B as a bait (62). We validated this interaction by co-immunoprecipitation with both EBNA3B and EBNA3A (Supplementary Figure S1). Both CENPJ and EIF3F were previously identified in a Y2H screen using EBNA3C as a bait (63). We validated these interactions with both EBNA3C and EBNA3A for CENPJ and with all three EBNA3s for EIF3F (Supplementary Figure S1). The interaction of both EBNA3A and EBNA3C with the CtBP1 co-activator has already been well characterized (13,14,17). We also found an interaction with CtBP2, a protein highly related to CtBP1 (80% homology) (64) (Supplementary Figure S1). Finally, EBNA3A was previously found, by co-immunoprecipitation, to form a complex with HSP40 (DNAJB1) in LCLs (28). The fact that we identified this cellular partner by Y2H suggests a direct interaction between the two proteins. Moreover, we show that EBNA3B and EBNA3C can also be found in a complex with DNAJB1 (Supplementary Figure S1). We also identified several interactors that are part of protein complexes previously shown to be targeted by the EBNA3s. This is the case for STIP1 (also called HOP for HSC70/HSP90-organizing protein) which facilitates the association of the HSP70/HSP90 chaperone complex (65): EBNA3A has previously been shown to interact with HSP70 and HSP90 (28). This is also the case for FBXO18 (F-box protein, helicase, 18) which forms a SCF (SKP1/Cullin/F-box) complex with human CUL1 and ROC1 (66): EBNA3C has previously been shown to interact with the SCF^SKP2^ complex (9,10). EBNA3C (and EBNA3A) could thus potentially interact with several different SCF complexes.

Most importantly, we identified several novel interactors which have functions linked to transcriptional regulation, either directly, as DNA-binding transcriptional regulators (ZBTB17, NFYC and MEOX1) or co-activators/repressors (CtBP2, MED25 and MCRS1), or indirectly as components of the ubiquitin/proteasome pathway (FBXO18, SUMO2, COPS5). Of these novel interactors we became particularly interested in ZBTB17, which is more frequently referred to as MIZ-1 (for MYC-interacting zinc-finger protein 1). MIZ-1 is a POZ-ZF factor that binds to transcription Inr in the core promoter of target genes of important regulators of the cell cycle i.e. *CDKN1A*, *CDKN2B* and *CDKN1C* (36,37). We thus decided to further characterize the interactions between the EBNA3 proteins and MIZ-1.

### All three EBNA3 proteins interact with the cellular protein MIZ-1 both *in cellulo* and *in vitro*

**Figure 1.**
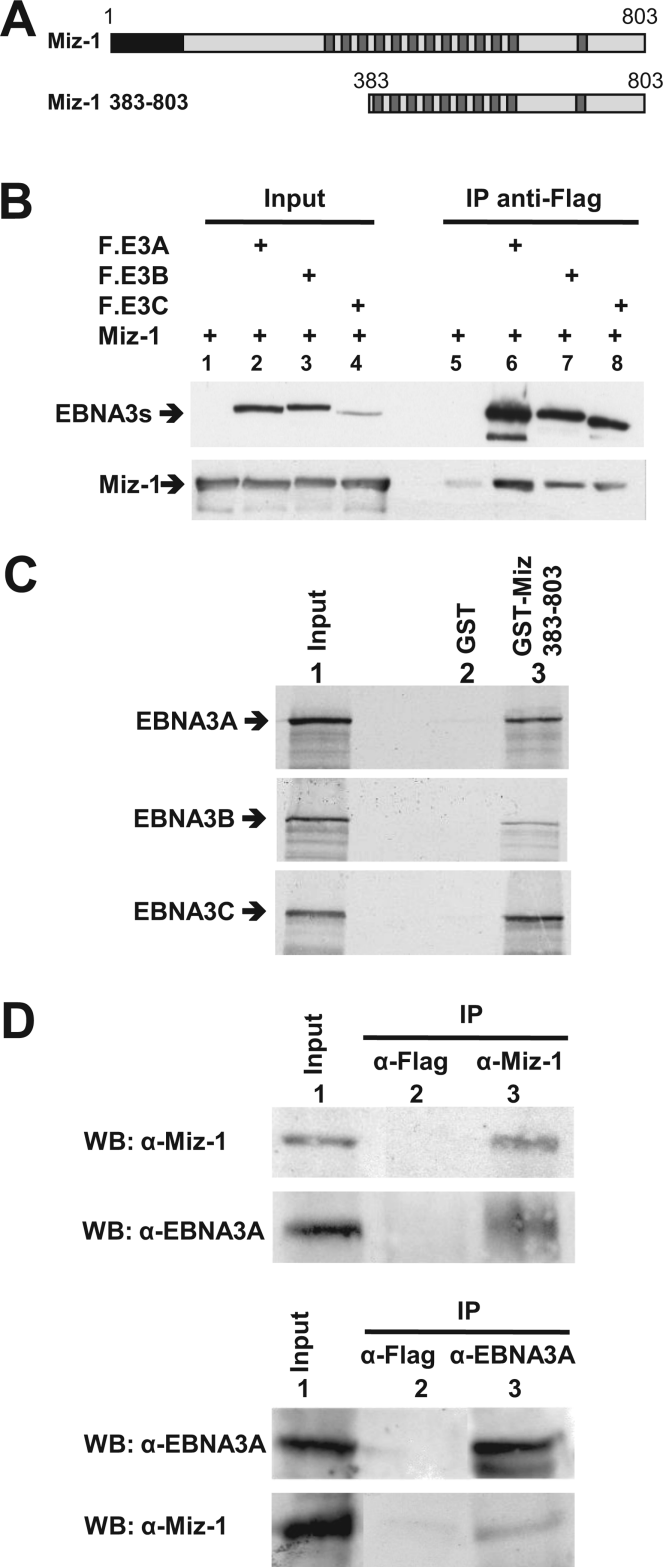
The EBNA3 proteins interact with the cellular protein MIZ-1. (**A**) Schematic representation of MIZ-1. The black box corresponds to the POZ domain of the protein and the dark gray boxes to zinc finger motifs. MIZ-1 380–803 corresponds to the protein expressed from the longest cDNA isolated in the Y2H screen. (**B**) Expression plasmids for Flag-EBNA3A (F.E3A), Flag-EBNA3B (F.E3B), Flag-EBNA3C (F.E3C) and MIZ-1 were transfected into HeLa cells as indicated. Cellular extracts were immunoprecipitated with an M2 anti-Flag mAb affinity gel and the immunoprecipitated complexes were analyzed by western blotting using an anti-Flag polyclonal antibody to detect the EBNA3 proteins (top panel) or an anti-Miz polyclonal antibody (bottom panel). Input corresponds to 8% of the cell extracts used for immunoprecipitation. (**C**) ^35^S-labeled EBNA3A, EBNA3B or EBNA3C were incubated with purified GST or GST-MIZ-1 383–803 bound to glutathione sepharose beads. The bound proteins were analyzed by SDS-PAGE and visualized by autoradiography. In lane 1, the equivalent of one-twelfth of the EBNA3A, EBNA3B or EBNA3C-expressing rabbit reticulocyte lysates used in each assay was loaded onto the gel. (**D**) EBNA3A or MIZ-1 were immunoprecipitated (IP) from a lymphoblastoid cell line protein extract using anti-EBNA3A or anti-MIZ-1-specific polyclonal antibodies as indicated or with an anti-Flag antibody as control. Immuno and co-immunoprecipitated EBNA3A and MIZ-1 proteins were analyzed by western blotting. Input corresponds to 25% of the cell extract used for immunoprecipitation.

Since all MIZ-1 cDNAs isolated from the screen contain only sequences coding for the CT region of the protein, each possessing a variable number of internal zinc finger repeats (nine for the longest) (Figure 1A), we first checked if the EBNA3s interact with full-length MIZ-1 (FL-MIZ-1). For this, we performed a co-immunoprecipitation assay using HeLa cells transfected with expression vectors for FL-MIZ-1 tagged with a Myc epitope, and each one of the EBNA3s tagged with a Flag epitope. As can be seen in Figure 1B, FL-MIZ-1 was specifically co-immunoprecipitated with all three EBNA3s.

To confirm that MIZ-1 interacts directly with the EBNA3 proteins we performed a GST-Pulldown assay using GST-MIZ-1 383–803 (Figure 1A) produced in bacteria and ^35^S-EBNA3 proteins *in vitro-*translated in reticulocyte lysates. As shown in Figure 1C, EBNA-3A, -3B and -3C are all efficiently retained on GST-MIZ-1 383–803 but not on GST alone, which strongly suggests a direct interaction between MIZ-1 and each of the EBNA3s.

We next decided to investigate more specifically the physical and functional interactions between EBNA3A and MIZ-1. By reciprocal co-immunoprecipitation assays (Figure 1D) we demonstrated that endogenous MIZ-1 and EBNA3A can form a complex under physiological conditions in a relevant cell model—i.e. lymphoblastoid cells immortalized by EBV.

### The N-terminal domain of EBNA3A interacts with two different domains within MIZ-1

**Figure 2.**
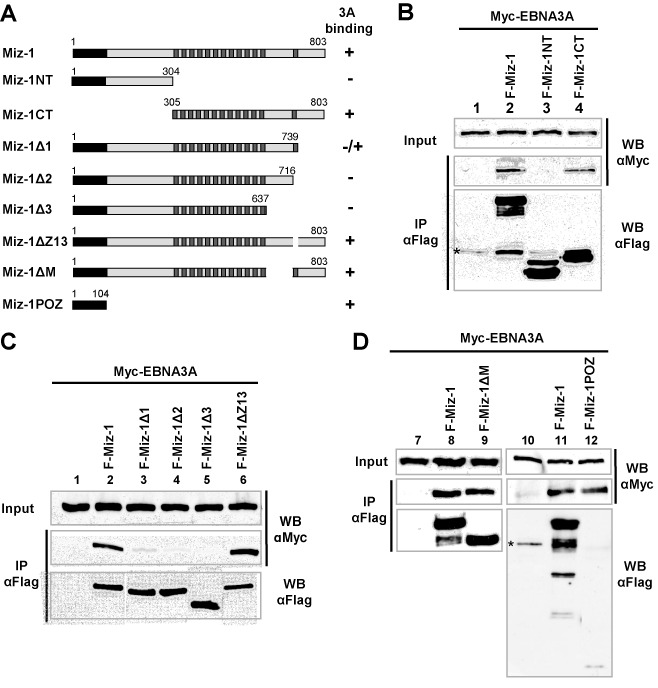
EBNA3A interacts with two distinct domains of Miz1. (**A**) Schematic representation of MIZ-1 and MIZ-1 deletion mutants. (**B**, **C** and **D**) Expression plasmids for Myc-EBNA3A and Flag-MIZ-1 or Flag-MIZ-1 deletion mutants were transfected into HeLa cells as indicated. Cellular extracts were immunoprecipitated with an M2 anti-Flag mAb affinity gel and the immunoprecipitated complexes were analyzed by western blotting using an anti-Flag polyclonal Flag antibody to detect MIZ-1 and the MIZ-1 deletion mutants or an anti-Myc 9E10 mAb to detect Myc-EBNA3A. Inputs correspond to 8% of the cell extract used for immunoprecipitation.

In order to precisely map the MIZ-1 domain(s) interacting with EBNA3A, we generated a series of MIZ-1 deletion mutants (Figure 2A) and performed co-immunoprecipitation assays. We first tested two complementary MIZ-1 mutants consisting of either the NT or the CT half of the protein. As can be seen in Figure 2B, both FL-MIZ-1 and MIZ-1CT interact with EBNA3A, whereas MIZ-1NT does not. This corroborates our finding that all MIZ-1 clones isolated in the Y2H screen contained the CT half of the protein albeit with a variable number of zinc finger motifs. We then tested a series of CT deletion mutants. As shown in Figure 2C, the mutants Δ1, Δ2 and Δ3 were all very inefficiently co-immunoprecipitated (lanes 3, 4, 5). By contrast, deletion of the zinc finger motif within the CT domain (mutant ΔZ13) had no effect (lane 6), neither did deletion of the domain between aa 638 and 716 previously shown to be required for the interaction with MYC (36) (Figure 2D, lane 9). Interestingly, we also found an interaction between the POZ domain of MIZ-1 and EBNA3A (Figure 2D, lane 12). This interaction appears to be as efficient as the interaction observed with FL-MIZ-1. This was unexpected since the mutants Δ1, Δ2, Δ3 and NT, that all contain the POZ domain do not interact efficiently with EBNA3A, suggesting that the capacity of the POZ domain to interact with EBNA3A is strongly perturbed in these deletion mutants. In conclusion, EBNA3A binds independently to both the NT POZ domain (aa 1–104) and the extreme CT (aa 739–803) of MIZ-1.

**Figure 3.**
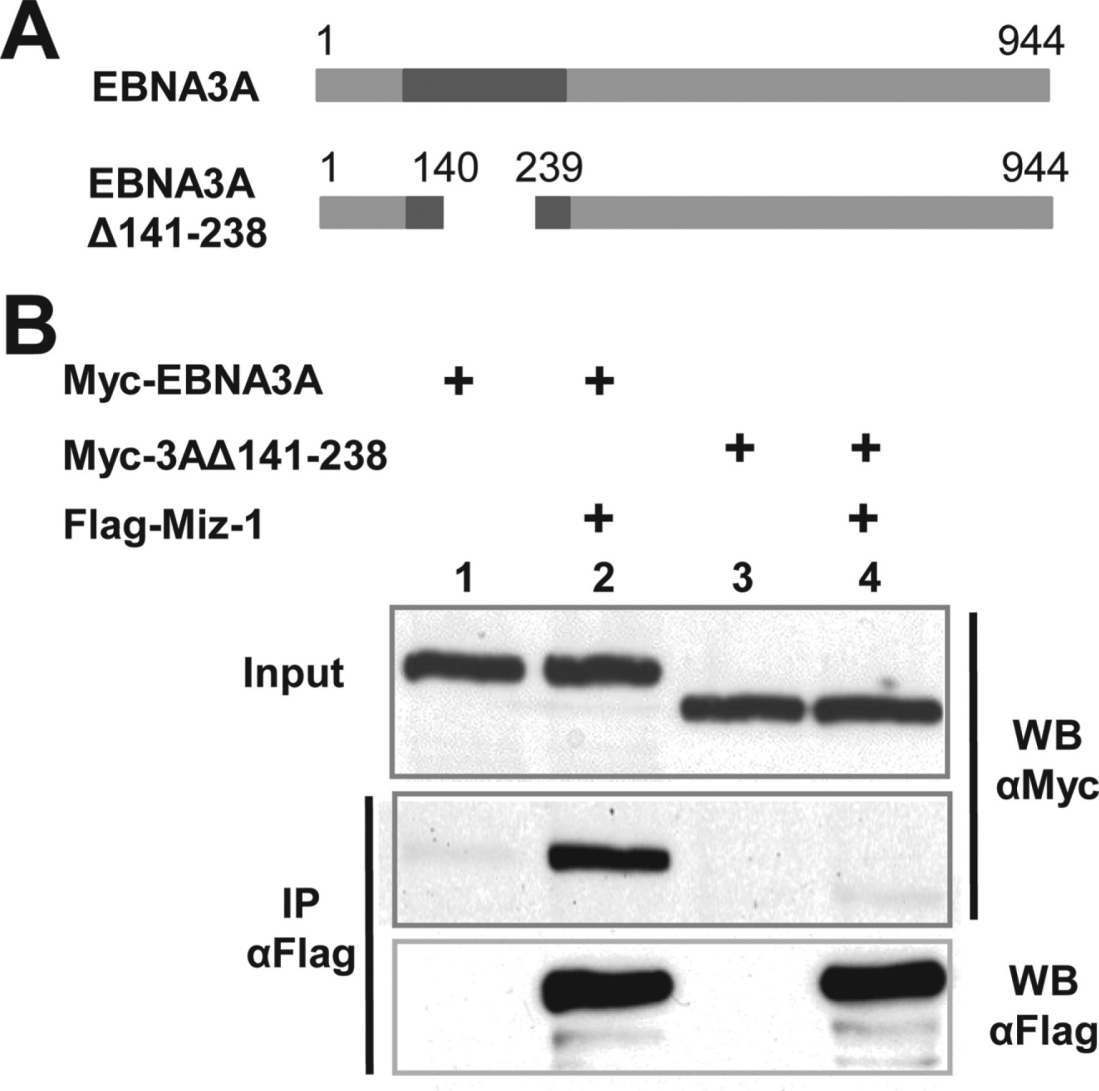
MIZ-1 interacts preferentially with the EBNA3 homology domain of EBNA3A. (**A**) Schematic representation of EBNA3A and EBNA3A deletion mutant. (**B**) Expression plasmids for Flag-MIZ-1 and Myc-EBNA3A or Myc-EBNA3A Δ141–238 were transfected into HeLa cells as indicated. Cellular extracts were immunoprecipitated with an M2 anti-Flag mAb affinity gel and the immunoprecipitated complexes were analyzed by western blotting using an anti-Flag polyclonal antibody to detect MIZ-1 or an anti-Myc 9E10 mAb to detect Myc-EBNA3A and Myc-EBNA3A Δ141–238. Input corresponds to 8% of the cell extract used for immunoprecipitation.

We then delimited the interaction domain of EBNA3A with MIZ-1. Because all three EBNA3s are able to interact with MIZ-1, we suspected the interaction domain to be localized within the homology domain of the EBNA3s (Figure 3A). We thus made use of an EBNA3A mutant with aa 141–238 deleted to test this hypothesis and indeed, co-immunoprecipitation of this deletion mutant with MIZ-1 was drastically reduced (Figure 3B). The importance of this domain for the interaction with both the MIZ-1 POZ domain and the MIZ-1 383–803 CT domain was then confirmed by GST-pulldown experiments (Supplementary Figure S2). Moreover, by using a series of NT and CT EBNA3A deletion mutants, we found that EBNA3A contacts the POZ domain of MIZ-1 via a minimum domain comprising aa 125–172 and that it also interacts with the CT of MIZ-1 via several regions—a major region is located in the first 224 NT amino acids of the protein and another in the CT half of the protein (Supplementary Figure S2).

### EBNA3A expression induces nuclear localization of MIZ-1

**Figure 4.**
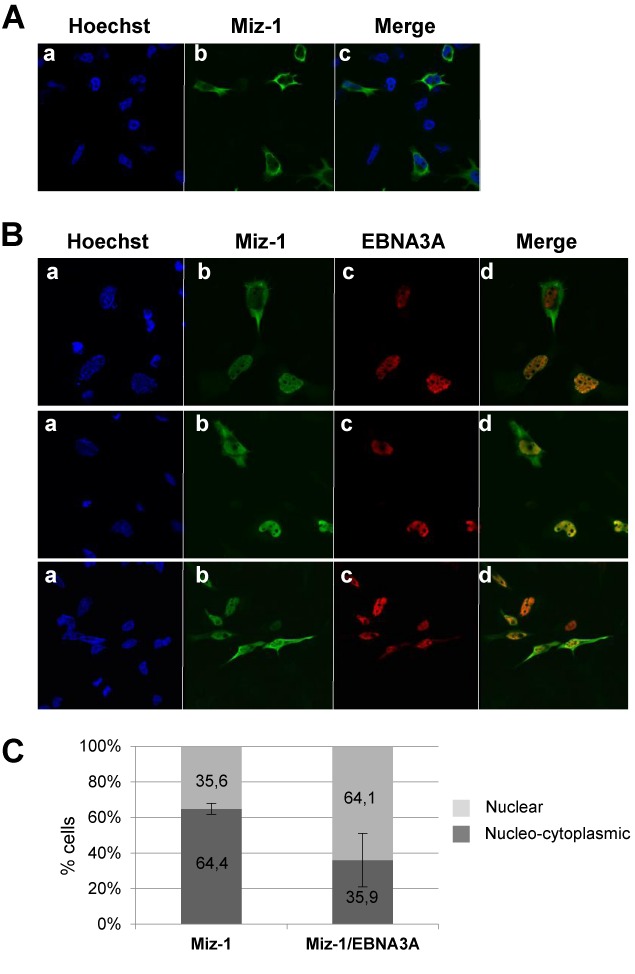
EBNA3A relocalizes MIZ-1 into the nucleus. Expression plasmids for Flag-MIZ-1 alone (**A**) or Flag-MIZ-1 together with Myc-EBNA3A were transfected into HeLa cells (**B** and **C**). Proteins were visualized by indirect immunofluorescence using an anti-Flag polyclonal Ab (for detection of MIZ-1) (panels b) and an anti-tag Myc polyclonal Ab (for detection of EBNA3A) (panels c). A Fluorolink Cy3-labeled goat anti-mouse IgG (H + L) antibody and an Alexa Fluor 488 goat anti-rabbit antibody were used as secondary antibodies respectively. Cell nuclei were stained with Hoechst 33258 (Sigma) (panels a). (C) Quantification of the number of cells in which MIZ-1 is strictly nuclear versus cytoplasmic/nucleo-cytoplasmic in cells transfected with an expression vector for MIZ-1 or in cells co-transfected with expression vectors for MIZ-1 and EBNA3A. In the latter case, only cells co-expressing EBNA3A together with MIZ-1 are taken into consideration. The results are the combination of two different experiments in which 1333 cells altogether were observed.

MIZ-1 was previously described to be present mostly in the cytoplasm of HeLa cells with only a small proportion of the protein being localized in the nucleus. However, in the presence of overexpressed MYC, most cells show an exclusive nuclear localization pattern of MIZ-1 (36). Since EBNA3A, like MYC, is a nuclear protein, we asked whether the expression of EBNA3A would also induce nuclear localization of MIZ-1. We thus co-transfected HeLa cells with an expression vector for MIZ-1 tagged with a Flag-epitope, together or not with an expression vector for EBNA3A tagged with a Myc-epitope. Immunofluorescence staining of the cells demonstrates that, as expected, MIZ-1 expressed alone is either fully cytoplasmic or nucleo-cytoplasmic in a majority of the cells (Figure 4A and C). By contrast, in the presence of EBNA3A, MIZ-1 is exclusively nuclear in most of the cells (Figure 4B and C). From these experiments, we conclude that co-expression of the two proteins results in their functional interaction and consequent migration of MIZ-1 from the cytoplasm to the nucleus.

### EBNA3A downregulates the expression of MIZ-1 target gene *CDKN2B* in LCLs

**Figure 5.**
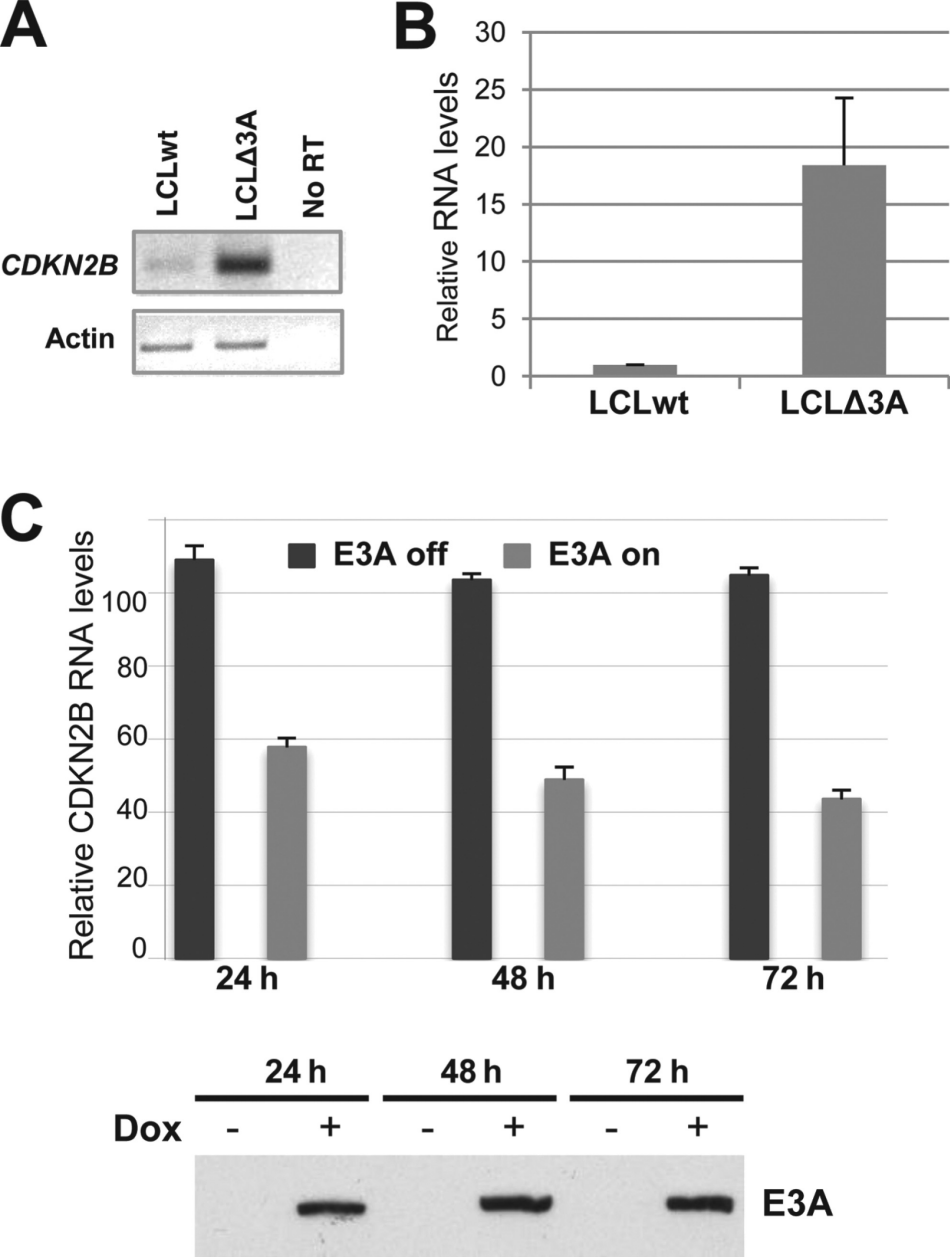
*CDKN2B* expression in lymphoblastoid cell lines is highly dependent on the presence or absence of EBNA3A. Analysis of *CDKN2B* mRNA expression levels from a normal LCL (wt) or an LCL established from the same donor but infected with an EBNA3A-deficient recombinant virus (D2 E3AmtB3) (LCLΔ3A) by either standard RT-PCR using actin mRNA as an internal control (**A**) or by real time RT-PCR (**B**). Histogram bars represent values relative to housekeeping gene *GAPDH*. Error bars represent standard deviation from four independent experiments. (**C**) ΔE3A-LCL^doxE3A^ cells were induced for EBNA3A expression by treatment with 200 ng/ml Dox for 24, 48 or 72 h or left untreated. The percentage of cells expressing the NGFR—a control gene expressed simultaneously with EBNA3A from a common bicistronic promoter—upon Dox treatment was evaluated by FACS to be 65% after 72 h. CDKN2B transcripts in total RNA were quantified by RTqPCR normalized to GAPDH RNA. EBNA3A protein levels were analyzed by western blotting (bottom panel). The data shown are derived from a single experiment but are representative of three experiments. Error bars represent standard deviation from three qPCR replicates.

Because MIZ-1 is implicated in the transcriptional regulation of the cyclin-dependent kinase inhibitor gene *CDKN2B* (encoding p15^INK4B^), we asked whether EBNA3A can modulate the expression of this gene. For this, we compared the level of transcript expressed in a B-LCL established with wt EBV (LCL_wt_) to that expressed in a LCL (LCL_Δ3A_) infected with an EBV mutant deficient for EBNA3A (E3AmtB mutant) (7). We first verified that the level of MIZ-1 protein was not affected by the presence or absence of EBNA3A in the LCLs (Supplementary Figure S3A). We then analyzed the levels of the *CDKN2B* transcript by standard and quantitative RT-PCR (Figure 5A and B). Interestingly, the level of the *CDKN2B* transcripts was drastically reduced in the presence of EBNA3A. We confirmed these observations in several LCLs immortalized independently with the E3AmtB mutant as well as LCLs obtained using a different donor and immortalized with a different type of EBNA3A-deficient mutant (mutant E3AmtA (7)). In all cases, we observed a similar decrease in *CDKN2B* RNA levels in the LCLs expressing EBNA3A compared to their counterpart infected with EBNA3A-deficient viruses (Supplementary Figure S3B). Finally, we analyzed the repression kinetics of *CDKN2B*. For this we made use of a LCL that expresses EBNA3A in a doxycycline (Dox) dependent manner (ΔE3A-LCL^doxE3A^) (31). As can be seen in Figure 5C, the transcript levels of *CDKN2B* fell rapidly—within 24 h—upon EBNA3A induction. Interestingly, we also observed a downregulation in the expression of *CDKN1C* and *CEBPD*—two genes that have previously been reported to be directly regulated by Miz-1 (41,67,68)—in the presence of EBNA3A (Supplementary Fig S4). In the case of *CDKN1A*—also a well documented target gene of Miz-1—we only observed a marginal, but reproducible, increase (Supplementary Figure S4). Moreover, in a recent paper Yenamandra et al. (69), using these same LCLs, found a 8-fold decrease in the level of *CDKN1A* transcripts in the presence of EBNA3A, which supports a role for EBNA3A in *CDKN1A* regulation. From these results we conclude that EBNA3A can repress transcription of MIZ-1 target genes.

**Figure 6.**
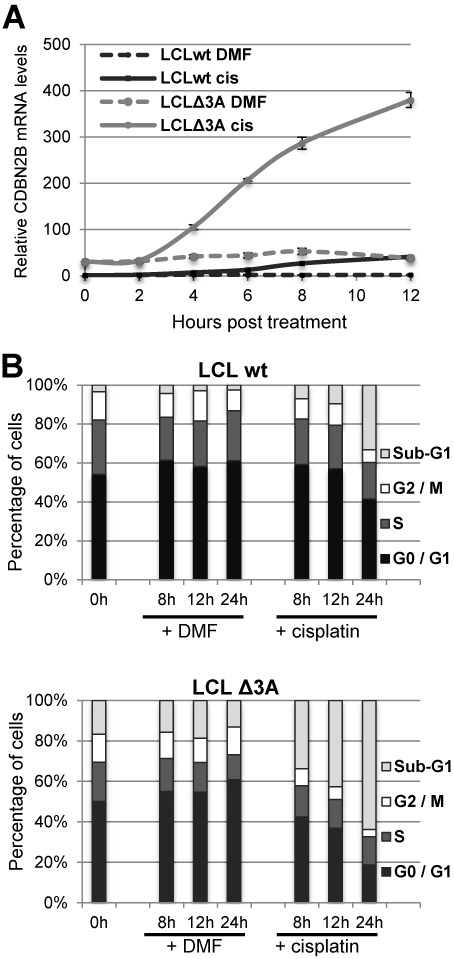
EBNA3A down-regulates CDKN2B's transcript expression levels in cells treated with cisplatin. LCL_wt_ and LCL_Δ3A_ were treated with 33 μM cisplatin or an equivalent amount of carrier (DMF). Cell aliquots were collected at 0, 2, 4, 6, 8 and 12 h for RNA analysis and at 0, 8, 12 and 24 h for cell cycle analysis. (**A**) CDKN2B transcripts in total RNA were quantified by RTqPCR normalized to GAPDH RNA. (**B**) Cells were stained with propidium iodide and analysed by FACS. The data shown are derived from a single experiment but are representative of three experiments. Error bars represent standard deviation from three qPCR replicates.

However, the above experiments were made using normal proliferating cells in which the level of *CDKN2B* transcript is expected to be low. By contrast, *CDKN2B* expression is known to be activated at the transcriptional level in a MIZ-1-dependent manner in response to UV irradiation, DNA damage or treatment with TGFβ (39,70,71). In order to test the impact of EBNA3A in conditions where *CDKN2B* expression is activated, we used cisplatin as a DNA-damage inducing agent. Cell cycle analysis revealed that treatment with cisplatin of either LCL_wt_ or LCL_Δ3A_ results in cell growth arrest and apoptosis as previously reported (72,73), but the response to the cisplatin treatment is clearly delayed in the case of LCL_wt_ compared to LCL_Δ3A_ (Figure 6B). In parallel, quantification by RTqPCR (Figure 6A) of *CDKN2B* transcript levels in the case of LCL_Δ3A_ over a 12 h time course period, shows a very rapid increase—within 4 h—in *CDKN2B* transcript levels. The maximum—a 10-fold increase—is reached after 12 h, beyond which the state of the cells did not allow further RNA quantification. In the case of LCL_wt_, the levels of the *CDKN2B* transcript are also induced following treatment with cisplatin—with a pattern similar to that observed in LCL_Δ3A_—but the levels remain very low, staying in the range observed in LCL_Δ3A_ before treatment with cisplatin.

Taken together, these results indicate that EBNA3A efficiently represses *CDKN2B* expression and that in conditions where this expression is induced by stimuli such as DNA-damage, this repression allows the retention of low levels of *CDKN2B* transcript compatible, at least to a certain extent, with cell survival.

### EBNA3A represses MIZ-1-mediated activation of a minimal *CDKN2B* gene promoter and interacts with a specific MIZ-1 target DNA probe containing an Inr sequence

**Figure 7.**
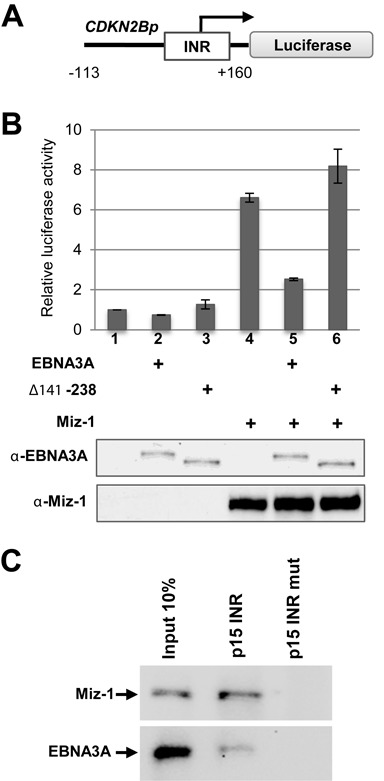
EBNA3A activates the *CDKN2B* minimal promoter and specifically binds the INR in association with MIZ-1. (**A**) Schematic representation of the *CDKN2B*p Luciferase reporter construct. The Firefly luciferase reporter gene was placed under the control of a −113/+160 bp fragment from the human *CDKN2B* promoter. INR: Initiator element. The arrow indicates the start of the transcription. (**B**) EBNA3A activates the *CDKN2B* minimal promoter. The *CDKN2B*p-Luc construct was transfected into HeLa cells, together with a CMV-renilla LUC plasmid as internal control and expression plasmids for EBNA3A or EBNA3A Δ141–238 and MIZ-1 as indicated in the figure. Results are plotted relative to the co-transfected CMV-renilla-LUC plasmid. Error bars represent standard deviations from three replicate assays. Protein expression levels were controled by western blotting using an anti-EBNA3A or an anti-MIZ-1 antibody for one representative experiment (bottom panel). (**C**) EBNA3A in association with MIZ-1 binds an oligonucleotide carrying the INR sequence. Whole LCLwt extracts were subjected to precipitation, using the *CDKN2B* promoter oligonucleotide—or a mutated control oligonucleotide that does not bind MIZ-1—and examined for the indicated proteins.

We next wanted to determine whether the repression effect of EBNA3A on *CDKN2B* expression was directly linked to MIZ-1 regulation of the *CDKN2B* promoter. To this end, we used a reporter construct in which the Firefly luciferase coding sequence was placed under the control of a minimal *CDKN2B* promoter (−113/+160). This contains an Initiator element (Inr) previously shown to recruit MIZ-1 to the promoter by direct binding of the protein to this sequence (37) (Figure 7A). As shown in Figure 7B, the reporter construct was activated by MIZ-1 in HeLa cells and this activation was efficiently repressed by EBNA3A but not by EBNA3A Δ141–238, a deletion mutant whose interaction with MIZ-1 is drastically reduced. These results indicate that EBNA3A specifically represses *CDKN2B* transcriptional activation by MIZ-1.

We then used an oligonucleotide precipitation assay to address whether MIZ-1 can recruit EBNA3A to the promoter of *CDKN2B*. For this, whole cell extracts of an LCL_wt_, endogenously expressing MIZ-1 and EBNA3A, were incubated with biotinylated double-stranded oligonucleotide containing the Inr sequence of the human *CDKN2B* promoter (or a control oligonucleotide with four bases mutated) (Supplementary Table S1). Bound proteins were precipitated using streptavidin-coated beads. As shown in Figure 7B, both MIZ-1 and EBNA3A were precipitated with the Inr oligonucleotide, but not with the mutated oligonucleotide, strongly suggesting that MIZ-1 recruits EBNA3A to its specific binding site on the DNA.

### Repression of *CDKN2B* expression by EBNA3A is associated with an increase in H3K27me3 at the *CDKN2B* promoter

**Figure 8.**
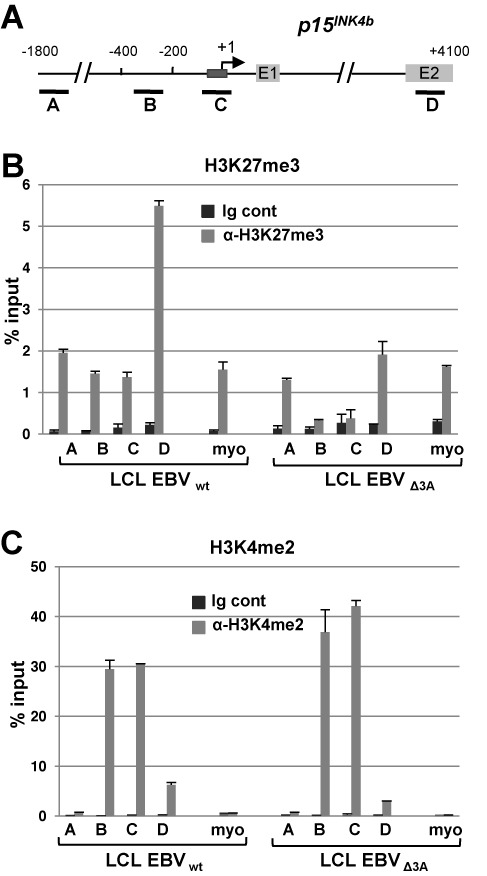
Epigenetic regulation of the *CDKN2B* locus by EBNA3A. (**A**) Schematic representation of the *CDKN2B* promoter region depicting the location of the fragments amplified in the ChIP-qPCR assays. The dark gray box represents the initiator element, the light gray boxes, the first two *CDKN2B* (*p15INK4b*) exons. (**B** and **C**) Comparative ChIP-qPCR analysis between LCLs transformed with either wt EBV or ΔEBNA3A EBV, quantifying the H3K27me3 (B) or the H3K4me2 (C) across the *CDKN2B* locus. The level of immunoprecipitated chromatin is expressed as a percentage of the input chromatin. Primers that amplify the promoter of inactive myoglobin (myo) were used as a control. Experiments were performed twice with two independent chromatin preparations.

EBNA3 proteins have previously been found to trigger the recruitment of polycomb repressive complex 2 (PRC2) core subunits and the trimethylation of H3K27 around both the *BCL2L11* promoter (27) and the *CDKN2A* locus encoding p16^INK4a^ and p14^ARF^ (17,16). We thus investigated the variation in H3K27 and H3K4 methylation levels at the *CDKN2B* promoter, depending on the presence or absence of EBNA3A. For this, ChIP, coupled with quantitative PCR (qPCR) assays directed across the *CDKN2B* locus (Figure 8A), were performed using EBV_wt_ and EBV_Δ3A_ LCLs. As can be seen in Figure 8B, the presence of EBNA3A correlates with a net increase in the repressive H3K27me3 mark, specifically centered on the site of the Inr motif. Concomitantly, as shown in Figure 8C, the H3K4 methylation was only marginally changed, which is reminiscent of what has also been observed in the case of the *BCL2L11* and *CDKN2A* loci (27,17,16). From these experiments we conclude that the repression mechanism of *CDKN2B* expression by EBNA3A is due, at least partially, to epigenetic modifications, in particular the increase in H3K27 tri-methylation similar to what has been observed for the *BCL2L11* and *CDKN2A* promoter regions.

### EBNA3A represses MIZ-1-mediated activation of the *CDKN2B* promoter in a CTBP-independent manner

**Figure 9.**
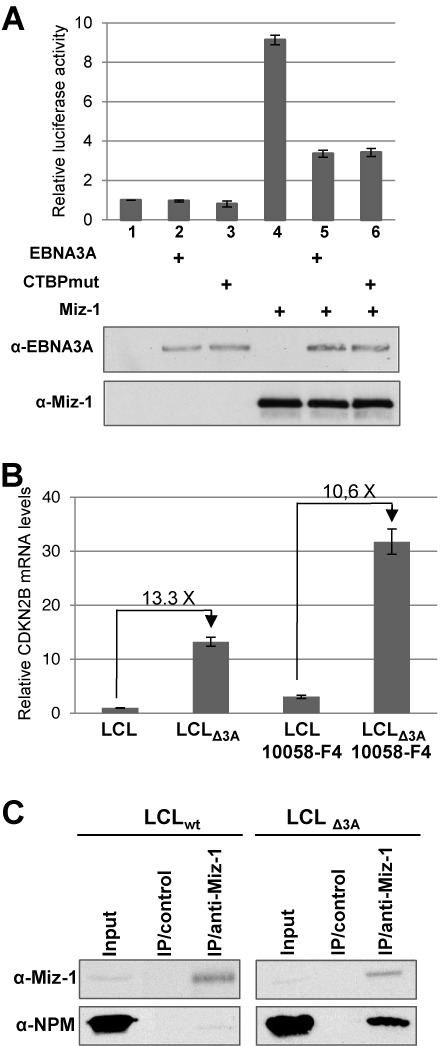
Mechanims of inhibition by EBNA3A of the MIZ-1-dependent activation of the *CDKN2B* transcription. (**A**) EBNA3A-dependent downregulation of *CDKN2B* expression is independent of CtBP. The CDKN2p-Luc construct was transfected into HeLa cells, together with a CMV-renilla LUC plasmid as internal control and expression plasmids for EBNA3A or EBNA3A-CtBPmut and MIZ-1 as indicated in the Figure. Results are plotted relative to the co-transfected CMV-renilla-LUC plasmid. Error bars represent standard deviations from three replicate assays. Protein expression levels were controled by western blotting using an anti-EBNA3A or an anti-MIZ-1 antibody for one representative experiment (bottom panels). (**B**) EBNA3A-dependent downregulation of *CDKN2B* expression is independent of MYC. The LCL_wt_ and LCL_Δ3A_ were treated or not with the 10058-F4 MYC inhibitor. Quantification of *CDKN2B* mRNA levels was performed by real time RT-PCR. Histogram bars represent values relative to housekeeping gene *GAPDH*. Error bars represent standard deviation from three replicate assays based on two different cDNAs. (**C**) EBNA3A inhibits MIZ-1 interaction with its coactivator Nucleophosmin. MIZ-1 was immunoprecipitated from eithe the LCL_wt_ or the LCL_Δ3A_ with an anti-MIZ-1 specific polyclonal antibody or with an unrelated antibody. Immunoprecipitated MIZ-1 and co-immunoprecipitated Nucleophosmin (NPM) proteins were analyzed by western blotting. Inputs correspond to 5% of the cell extract used for immunoprecipitation.

Although we found a good correlation between the presence of EBNA3A and epigenetic modifications, these modifications do not likely account for the very rapid transcription shut-down of *CDKN2B* following Dox-induced expression of EBNA3A. Since EBNA3A has previously been shown to repress transcription by recruiting the co-repressor CTBP1 (13), we first asked whether the interaction of EBNA3A with CTBP1 was required for the repression of MIZ-1-dependent transcriptional activation. As EBNA3A has been shown to interact with CTBP1 via two motifs, ALDLS and VLDLS, located in the CT part of EBNA3A, we generated an EBNA3A mutant with these two motifs mutated (EBNA3A-CTBPmut) (13). We then compared the capacity of this mutant to inhibit MIZ-1-dependent transcriptional activation of our reporter construct with that of wild-type EBNA3A. As can be seen Figure 9A, EBNA3A-CTBPmut represses MIZ-1 transcriptional activation as efficiently as EBNA3A, indicating that CTBP binding to EBNA3A is not required for its downregulation of the *CDKN2B* promoter.

### EBNA3A downregulation of *CDKN2B* expression is independent of MYC

Regulation by MIZ-1 of Inr-containing promoters involves a complex network of interactions with multiple cellular proteins acting either as co-activators or co-repressors. Among these, MYC has been shown to be an important down-regulator of *CDKN2B* expression via direct interaction with MIZ-1. Since the EBNA3 proteins have previously been shown to inhibit *MYC* transcription (61) and, at least EBNA3C, reported to directly interact with MYC (74), we investigated whether EBNA3A-induced down-regulation of *CDKN2B* expression could be due to an indirect effect via MYC. To examine this eventuality, we quantified the level of expression of *CDKN2B* in LCLs treated or not with a specific inhibitor of MYC: (Z,E)-5-(4-Ethylbenzylidine)-2-thioxothiazolidin-4–1 or 10058-F4 (75). As shown in Figure 9B and as expected, following treatment with 10058-F4 of both LCLs, infected with EBV_wt_ or EBV_Δ3A_ respectively, the level of *CDKN2B* mRNA is increased (between 2.4 to 3×). Interestingly, the difference in the *CDKN2B* transcript levels between normal LCL and LCL infected with EBV_Δ3A_ remains very similar, whether the cells are treated or not with 10058-F4. This indicates that down-regulation of *CDKN2B* expression by MYC or EBNA3A is mediated by independent mechanisms.

### EBNA3A inhibits MIZ-1 interaction with its co-activator Nucleophosmin (NPM)

Finally, several cellular co-activators of MIZ-1 have been previously identified, like NPM/B23 (38) which interacts with the POZ domain of MIZ-1. Since EBNA3A also interacts with MIZ-1's POZ domain, we asked whether EBNA3A could interfere with MIZ-1/NPM interaction. To explore this hypothesis, we co-immunoprecipitated MIZ-1/NPM complexes from LCLs infected either with EBV_wt_ or with EBV_Δ3A_. As shown in Figure 9C, MIZ-1 was expressed and immunoprecipitated in similar amounts from both LCLs. Interestingly, NPM was efficiently co-immunoprecipitated from the EBV_Δ3A_-infected LCL, whereas very little NPM was found associated with MIZ-1 in the LCL infected with EBV_wt_ although the overall level of NPM was identical in both cell lines. It should be noted that NPM itself does not appear to interact with EBNA3A (Supplementary Data Figure S4). These results strongly suggest that EBNA3A inhibits MIZ-1-mediated transcriptional activation by preventing the interaction of MIZ-1 with its coactivator, NPM.

## DISCUSSION

In this study we have developed a large-scale Y2H screen to identify the main cellular partners of the EBNA3 proteins. We first identified the majority of the previously well-characterized cellular partners of the EBNA3s, which validated the efficiency of our screen. In addition to these already reported interactors, we have identified 22 new cellular partners for the EBNA3s. Most importantly, among the newly identified partners of the EBNA3s, several—MCRS1, MEOX1, MED25, NF-YC and ZBTB17 (MIZ-1)—are known to play direct roles in transcriptional regulation. MCRS1 is a putative regulatory component of the chromatin remodeling INO80 complex, which is involved in transcriptional regulation, DNA replication and DNA repair (76). MEOX1 has recently been found to activate transcription of *CDKN2A* (encoding p16^INK4A^) in endothelial cells in a DNA-binding-dependent manner (77). Interestingly, *CDKN2A* is an important target of both EBNA3A and EBNA3C in LCLs (7,6,17,16). It is thus tempting to postulate that transcriptional repression of *CDKN2A* expression by the EBNA3s involves an interaction with MEOX1. MED25 is a component of the Mediator complex, a large polypeptide complex consisting of about thirty polypeptides involved in the transcriptional regulation of nearly all RNA polymerase II (RNAP II)-dependent genes. Mediator forms a bridge between gene-specific transcription factors and the RNAP II machinery via direct interactions between specific mediator subunits and specific transcription factors. MED25 in particular has been found to interact with HSF, HNF4, SOX9 cellular factors and VP16 from herpes simplex virus (for a review see (78)). Interaction between the EBNA3s and the mediator complex via MED25 is thus of particular interest and needs to be further explored. NF-YC is one of the subunits of Nuclear transcription factor Y (NF-Y), a ubiquitous heteromeric transcription factor composed of three subunits - NF-YA, NF-YB and NF-YC - that binds CCAAT elements in the core promoter of many genes (for a review see (79)). Interestingly, it has been shown that EBNA3C and EBNA3A cooperate to repress expression of the proapoptotic tumor-suppressor BCL2L11 (BIM) in BL cell lines (15) and that EBNA3C can be recruited to the *BIM* promoter proximal to the transcription start site (TSS) (80). Moreover, it has recently been shown, in rat sympathetic neurons, that the *BCL2L11* core promoter is regulated by NF-Y via a CCAAT box proximal to the TSS (81). Since the CCAAT box found in the rat *BCL2L11* promoter is conserved in the human gene promoter, it is tempting to hypothesize that the EBNA3s are specifically recruited onto the *BCL2L11* promoter by interacting with NF-Y bound to the CCAAT box. Work is currently under progress in our laboratory to determine whether this is indeed the case. Finally, MIZ-1 is a transcriptional regulator involved in the expression of many genes involved in cell control (*CDKN1A*, *CDKN2B* and *CDKN1C* for example) or apoptosis regulation (*BCL2*). By interacting with such crucial factors, the EBNA3 proteins are likely to induce a complete reprogrammation of transcription in B cells following infection by EBV.

Given the important role of MIZ-1 in the control of cell proliferation, we decided to focus our attention on the functional role of this particular interaction in EBV-driven B-cell proliferation. Following our original finding that MIZ-1 interacts with the three EBNA3 proteins, we subsequently studied more specifically the interaction between MIZ-1 and EBNA3A. EBNA3A interacts both with the NT POZ domain of MIZ-1 and with a CT domain located between aa 740–803. The main interaction domain in EBNA3A is localized within the NT and corresponds to a ‘homology domain’—from aa 90–310 for EBNA3A—common to all three EBNA3s. However, the precise interacting sub-regions involved in contacting respectively the POZ domain of MIZ-1 and its CT region are likely to be different since although interaction with the CT of MIZ-1 is completely lost in deletion mutant, EBNA3A 1–172, this mutant still interacts with the POZ domain. Thus, interaction between the two proteins appears to be particularly complex and will require structural studies in order to be characterized further.

One of the best characterized target genes of MIZ-1 is the cyclin-dependent kinase inhibitor (CDKI) p15^INK4B^ (*CDKN2B*) (37,40). Comparison of the *CDKN2B* transcript levels clearly shows that EBNA3A presence correlates with a lower level of *CDKN2B*. However, although we obtained similar results with different combinations of LCLs—established from different donors and infected with either wild-type or one of two different types of EBNA3A-mutated viruses—we were concerned by the fact that *CDKN2B* had not been reported to be an EBNA3A-regulated gene in previous microarray studies (7). In order to clarify the question, we used conditions in which the expression level of the gene was induced by treatment of the cells with the DNA-damaging agent cisplatin, and were able to confirm our original observation of a significant downregulation of the *CDKN2B* transcript level in the presence of EBNA3A. Thus by keeping a low level of *CDKN2B* transcripts, EBNA3A probably participates in delaying cell cycle arrest following DNA damage.

By using a luciferase reporter assay in which MIZ-1 activates expression of the luciferase gene placed under the control of a minimal *CDKN2B* promoter containing mainly the Inr known to directly bind MIZ-1, we found that EBNA3A—but not a mutant of EBNA3A altered for its capacity to interact with MIZ-1—was able to antagonize Miz-dependent activation of the *CDKN2B* promoter. This suggests that interaction with MIZ-1 is indeed directly responsible for the downregulation of the *CDKN2B* transcript level observed in the LCLs. Moreover, by using an oligo-pull down assay, we found that EBNA3A specifically associates with MIZ-1 bound on its specific Inr recognition sequence, strongly suggesting that MIZ-1 recruits EBNA3A to the DNA. Similar results were obtained for EBNA3C in these two assays (data not shown). In the event, however, we were not able to obtain convincing ChIPs of the *CDKN2B* promoter region—neither with an anti-EBNA3A nor with anti-EBNA3C antibodies—whereas we efficiently immunoprecipitated the ADAM28/ADAMDEC1 intergenic region, previously described as a target for both EBNA3A and EBNA3C (29,33). However, in a recent paper, Skalska et al. (17), using a LCL established with a recombinant EBV expressing an EBNA3C protein tagged with a Flag epitope at its CT, and an anti-Flag antibody, succeeded in performing ChIP experiments that demonstrate the presence of EBNA3C immediately proximal to all three *CDKN2B, ARF* and *INK4a* (these latter two being jointly referred to as *CDKN2A*) transcription starts. This latter result strongly supports the idea that the EBNA3s are indeed recruited by MIZ-1 to the *CDKN2B* promoter proximal region. That we could not detect this promoter region in our ChIP assays using antibodies against both EBNA3A and EBNA3C, suggests that the epitopes recognised by these antibodies are not likely to be accessible in the context of the *CDKN2B* promoter.

Finally, we were interested in investigating the mechanisms involved in the repression of MIZ-1 transcriptional activation. Since the EBNA3s have previously been shown to be involved in inducing epigenetic modifications at various promoters (27,16,31), we investigated the impact of the absence of EBNA3A on the methylation state of the chromatin at the *CDKN2B* locus and accordingly observed a decrease in the amount of H3K27me3 but no major change in the amount of H3K4me2. This suggests that, similar to what has been described for the *BCL2L11* (27) promoter, EBV downregulates *CDKN2B* expression likely by triggering recruitment of the polycomb repressive complex 2 (PRC2) leading to trimethylation of histone H3 lysine 27 at the *CDKN2B* locus. Furthermore, in accordance to what was previously observed in the case of the *BCL2L11* promoter, absence of only EBNA3A protein is sufficient to completely inhibit H3K27 methylation at the *CDKN2B* promoter. Whether EBNA3C cooperates with EBNA3A for triggering epigenetic modifications at the *CDKN2B* promoter—in a similar way to what has been found in the case of the *BCL2L11* promoter—remains to be investigated.

Besides this role of the EBNA3s in triggering epigenetic modification, another way in which EBNA3A could inhibit MIZ-1-dependent transcriptional activation would be to interfere with MIZ-1 binding to its coactivator(s). Here we demonstrate that EBNA3A inhibits MIZ-1 interaction with its coactivator, NPM. This result is in accordance with our mapping of the EBNA3A interaction domain to the POZ domain of MIZ-1 which happens also to be the interaction domain with NPM (38). Taken together, these results suggest two levels of repression of MIZ-1's target promoter *CDKN2B* by EBNA3A : first, EBNA3A binding to MIZ-1 displaces MIZ-1 coactivator NPM, thus inhibiting MIZ-1-dependent transcriptional activation. That this event occurs promptly following the expression of EBNA3A, was revealed in the experiments where EBNA3A expression was induced by Dox. Second, EBNA3A—possibly in cooperation with EBNA3C—triggers the recruitment of PRC2 as has been previously described for the *BCL2L11* promoter. Interestingly, another link between EBV and NPM has been recently documented in a paper by Liu *et al.* (82). Indeed, the authors show that NPM expression is induced in EBV-infected cells and they demonstrate an essential role for NPM in chaperoning EBNA2 recruitment to the latency-associated membrane protein 1 (LMP1) promoter. Thus NPM appears to be essential for the activation of the EBV genes expressed during latency and consequently for EBV-driven B-cell proliferation. Paradoxically, by acting as a coactivator of MIZ-1, NPM also has the capacity to enhance expression of the cell cycle inhibitors *CDKN2B* and *CDKN1A* (38). By inhibiting the interaction between NPM and MIZ-1, the EBNA3s could thus play an important role by counteracting the inhibiting effect of NPM on cell proliferation.

Although the EBNA3 proteins were for long recognized for their capacity as transcriptional regulators, until recently only RBP-J was consistently reported to be a DNA-binding protein that directs the EBNA3s to specific promoter elements. On the other hand, ChIP-sequencing (ChIP-seq) analyses identified up to 13 000 genomic sites for the EBNA3s (32,33), of which only a few (around 16%) coincide with RBP-J binding sites. Moreover, a majority of EBNA3 binding sites are localized in enhancer regions and it has been suggested that EBNA3 proteins regulate transcription by modulating enhancer-promoter loop formation (33). However, although ChIP-seq analysis revealed frequent co-occupancy with a handful of cellular transcription factors, the cellular proteins allowing the formation of such loop have not as yet been defined (32,33). Here we describe, for the first time, the interaction of the EBNA3s with MIZ-1, a transcription factor that binds Inrs present in the core promoters of genes. This factor is thus a very good candidate to participate—as a promoter binding factor—in the enhancer-promoter loop formation promoted by the EBNA3s.

Finally, our findings are in line with the idea of a central role for MIZ-targeting in tumorigenesis. It was recently proposed that the functional interaction between MYC and MIZ-1 mediates resistance to anti-mitogenic signals and that in the context of MYC deregulation this interaction could play a central role in tumorigenesis (83). That MIZ-1 potentially plays an important role in the course of oncogenesis is reinforced by the notion that MIZ-1 is targeted by oncovirus proteins such as the EBV EBNA3s as discussed in this paper and also by the Human Papilloma virus type 16 E7 oncoprotein (84). It is interesting to note that among the different B cell lymphoma and lymphoproliferations associated with EBV, the EBNA3s are usually not expressed in Burkitt's lymphoma cells where a translocation involving the MYC gene results in MYC overexpression, whereas the EBNA3s expression—at least that of EBNA3A and EBNA3C—is central for efficient proliferation of EBV growth-transformed LCLs. It is thus tempting to postulate that MYC overpexression in Burkitt's lymphoma and EBNA3s expression in EBV-associated lymphoproliferations fulfil—via MIZ-1—similar functions important for continuous cell proliferation.

## SUPPLEMENTARY DATA

Supplementary Data are available at NAR Online.

SUPPLEMENTARY DATA

## References

[1] Crawford D.H. (2001). Biology and disease associations of Epstein-Barr virus. Philos. Trans. R. Soc. Lond. B. Biol. Sci..

[2] Kieff E., Rickinson A.B., Fields Knipe & Howley (2007). Epstein Barr virus and its replication. Fields Virology.

[3] Cai X., Schafer A., Lu S., Bilello J.P., Desrosiers R.C., Edwards R., Raab-Traub N., Cullen B.R. (2006). Epstein-Barr virus microRNAs are evolutionarily conserved and differentially expressed. PLoS Pathog..

[4] Tomkinson B., Robertson E., Kieff E. (1993). Epstein-Barr virus nuclear proteins EBNA-3A and EBNA-3C are essential for B-lymphocyte growth transformation. J. Virol..

[5] Maruo S., Johannsen E., Illanes D., Cooper A., Kieff E. (2003). Epstein-Barr Virus nuclear protein EBNA3A is critical for maintaining lymphoblastoid cell line growth. J. Virol..

[6] Maruo S., Wu Y., Ishikawa S., Kanda T., Iwakiri D., Takada K. (2006). Epstein-Barr virus nuclear protein EBNA3C is required for cell cycle progression and growth maintenance of lymphoblastoid cells. Proc. Natl. Acad. Sci. U.S.A..

[7] Hertle M.L., Popp C., Petermann S., Maier S., Kremmer E., Lang R., Mages J., Kempkes B. (2009). Differential gene expression patterns of EBV infected EBNA-3A positive and negative human B lymphocytes. PLoS Pathog..

[8] Yi F., Saha A., Murakami M., Kumar P., Knight J.S., Cai Q., Choudhuri T., Robertson E.S. (2009). Epstein-Barr virus nuclear antigen 3C targets p53 and modulates its transcriptional and apoptotic activities. Virology.

[9] Knight J.S., Sharma N., Robertson E.S. (2005). Epstein-Barr virus latent antigen 3C can mediate the degradation of the retinoblastoma protein through an SCF cellular ubiquitin ligase. Proc. Natl. Acad. Sci. U.S.A..

[10] Knight J.S., Sharma N., Robertson E.S. (2005). SCFSkp2 complex targeted by Epstein-Barr virus essential nuclear antigen. Mol. Cell. Biol..

[11] Knight J.S., Sharma N., Kalman D.E., Robertson E.S. (2004). A cyclin-binding motif within the amino-terminal homology domain of EBNA3C binds cyclin A and modulates cyclin A-dependent kinase activity in Epstein-Barr virus-infected cells. J. Virol..

[12] Parker G.A., Crook T., Bain M., Sara E.A., Farrell P.J., Allday M.J. (1996). Epstein-Barr virus nuclear antigen (EBNA)3C is an immortalizing oncoprotein with similar properties to adenovirus E1A and papillomavirus E7. Oncogene.

[13] Hickabottom M., Parker G.A., Freemont P., Crook T., Allday M.J. (2002). Two non-consensus sites in the Epstein-Barr virus oncoprotein EBNA3A co-operate to bind the co-repressor CtBP. J. Biol. Chem..

[14] Touitou R., Hickabottom M., Parker G., Crook T., Allday M.J. (2001). Physical and functional interactions between the corepressor CtBP and the Epstein-Barr virus nuclear antigen EBNA3C. J. Virol..

[15] Anderton E., Yee J., Smith P., Crook T., White R.E., Allday M.J. (2008). Two Epstein-Barr virus (EBV) oncoproteins cooperate to repress expression of the proapoptotic tumour-suppressor Bim: clues to the pathogenesis of Burkitt's lymphoma. Oncogene.

[16] Skalska L., White R.E., Franz M., Ruhmann M., Allday M.J. (2010). Epigenetic repression of p16(INK4A) by latent Epstein-Barr virus requires the interaction of EBNA3A and EBNA3C with CtBP. PLoS Pathog..

[17] Skalska L., White R.E., Parker G.A., Sinclair A.J., Paschos K., Allday M.J. (2013). Induction of p16(INK4a) is the major barrier to proliferation when Epstein-Barr virus (EBV) transforms primary B cells into lymphoblastoid cell lines. PLoS Pathog..

[18] Marshall D., Sample C. (1995). Epstein-Barr virus nuclear antigen 3C is a transcriptional regulator. J. Virol..

[19] Bain M., Watson R.J., Farrell P.J., Allday M.J. (1996). Epstein-Barr virus nuclear antigen 3C is a powerful repressor of transcription when tethered to DNA. J. Virol..

[20] Waltzer L., Perricaudet M., Sergeant A., Manet E. (1996). Epstein-Barr virus EBNA3A and EBNA3C proteins both repress RBP-Jk-EBNA2-activated transcription by inhibiting the binding of RBP-Jk to DNA. J. Virol..

[21] Bourillot P.-Y., Waltzer L., Sergeant A., Manet E. (1998). Transcriptional repression by the Epstein-Barr virus EBNA3A protein tethered to DNA does not require RBP-Jk. J. Gen. Virol..

[22] Cludts I., Farrell P.J. (1998). Multiple functions within the Epstein-Barr virus EBNA-3A protein. J. Virol..

[23] Cotter M.A., Robertson E.S. (2000). Modulation of histone acetyltransferase activity through interaction of epstein-barr nuclear antigen 3C with prothymosin alpha. Mol. Cell. Biol..

[24] Subramanian C., Hasan S., Rowe M., Hottiger M., Orre R., Robertson E.S. (2002). Epstein-Barr virus nuclear antigen 3C and prothymosin alpha interact with the p300 transcriptional coactivator at the CH1 and CH3/HAT domains and cooperate in regulation of transcription and histone acetylation. J. Virol..

[25] Radkov S.A., Touitou R., Brehm A., Rowe M., West M., Kouzarides T., Allday M.J. (1999). Epstein-Barr virus nuclear antigen 3C interacts with histone deacetylase to repress transcription. J. Virol..

[26] Knight J.S., Lan K., Subramanian C., Robertson E.S. (2003). Epstein-Barr virus nuclear antigen 3C recruits histone deacetylase activity and associates with the corepressors mSin3A and NCoR in human B-cell lines. J. Virol..

[27] Paschos K., Parker G.A., Watanatanasup E., White R.E., Allday M.J. (2012). BIM promoter directly targeted by EBNA3C in polycomb-mediated repression by EBV. Nucleic Acids Res..

[28] Young P., Anderton E., Paschos K., White R., Allday M.J. (2008). Epstein-Barr virus nuclear antigen (EBNA) 3A induces the expression of and interacts with a subset of chaperones and co-chaperones. J. Gen. Virol..

[29] McClellan M.J., Khasnis S., Wood C.D., Palermo R.D., Schlick S.N., Kanhere A.S., Jenner R.G., West M.J. (2012). Downregulation of integrin receptor-signaling genes by Epstein-Barr virus EBNA 3C via promoter-proximal and -distal binding elements. J. Virol..

[30] Zhao B., Mar J.C., Maruo S., Lee S., Gewurz B.E., Johannsen E., Holton K., Rubio R., Takada K., Quackenbush J. (2010). Epstein-Barr virus nuclear antigen 3C regulated genes in lymphoblastoid cell lines. Proc. Natl. Acad. Sci. U.S.A..

[31] Harth-Hertle M.L., Scholz B.A., Erhard F., Glaser L.V., Dölken L., Zimmer R., Kempkes B. (2013). Inactivation of intergenic enhancers by EBNA3A initiates and maintains polycomb signatures across a chromatin domain encoding CXCL10 and CXCL9. PLoS Pathog..

[32] Jiang S., Willox B., Zhou H., Holthaus A.M., Wang A., Shi T.T., Maruo S., Kharchenko P.V., Johannsen E.C., Kieff E. (2014). Epstein-Barr virus nuclear antigen 3C binds to BATF/IRF4 or SPI1/IRF4 composite sites and recruits Sin3A to repress CDKN2A. Proc. Natl. Acad. Sci. U.S.A..

[33] McClellan M.J., Wood C.D., Ojeniyi O., Cooper T.J., Kanhere A., Arvey A., Webb H.M., Palermo R.D., Harth-Hertle M.L., Kempkes B. (2013). Modulation of enhancer looping and differential gene targeting by Epstein-Barr virus transcription factors directs cellular reprogramming. PLoS Pathog..

[34] Le Roux A., Kerdiles B., Walls D., Dedieu J.F., Perricaudet M. (1994). The Epstein-Barr virus nuclear antigens EBNA-3A, 3B, 3C repress EBNA-2-mediated transactivation of the viral terminal protein 1 gene promoter. Virology.

[35] Radkov S.A., Bain M., Farrell P.J., West M., Rowe M., Allday M.J. (1997). Epstein-Barr virus EBNA3C represses Cp, the major promoter for EBNA expression, but has no effect on the promoter of the cell gene CD21. J. Virol..

[36] Peukert K., Staller P., Schneider A., Carmichael G., Hanel F., Eilers M. (1997). An alternative pathway for gene regulation by Myc. EMBO J..

[37] Staller P., Peukert K., Kiermaier A., Seoane J., Lukas J., Karsunky H., Möröy T., Bartek J., Massagué J., Hänel F. (2001). Repression of p15INK4b expression by Myc through association with Miz-1. Nat. Cell Biol..

[38] Wanzel M., Russ A.C., Kleine-Kohlbrecher D., Colombo E., Pelicci P.G., Eilers M. (2008). A ribosomal protein L23-nucleophosmin circuit coordinates Mizl function with cell growth. Nat. Cell Biol..

[39] Seoane J., Pouponnot C., Staller P., Schader M., Eilers M., Massague J. (2001). TGFbeta influences Myc, Miz-1 and Smad to control the CDK inhibitor p15INK4b. Nat. Cell Biol..

[40] Seoane J., Le H.-V., Massagué J. (2002). Myc suppression of the p21(Cip1) Cdk inhibitor influences the outcome of the p53 response to DNA damage. Nature.

[41] Adhikary S., Eilers M. (2005). Transcriptional regulation and transformation by Myc proteins. Nat. Rev. Mol. Cell Biol..

[42] Phan R.T., Saito M., Basso K., Niu H., Dalla-Favera R. (2005). BCL6 interacts with the transcription factor Miz-1 to suppress the cyclin-dependent kinase inhibitor p21 and cell cycle arrest in germinal center B cells. Nat. Immunol..

[43] Saito M., Novak U., Piovan E., Basso K., Sumazin P., Schneider C., Crespo M., Shen Q., Bhagat G., Califano A. (2009). BCL6 suppression of BCL2 via Miz1 and its disruption in diffuse large B cell lymphoma. Proc. Natl. Acad. Sci. U.S.A..

[44] Basu S., Liu Q., Qiu Y., Dong F. (2009). Gfi-1 represses CDKN2B encoding p15INK4B through interaction with Miz-1. Proc. Natl. Acad. Sci. U.S.A..

[45] Piluso D., Bilan P., Capone J.P. (2002). Host cell factor-1 interacts with and antagonizes transactivation by the cell cycle regulatory factor Miz-1. J. Biol. Chem..

[46] Herkert B., Dwertmann A., Herold S., Abed M., Naud J.F., Finkernagel F., Harms G.S., Orian A., Wanzel M., Eilers M. (2010). The Arf tumor suppressor protein inhibits Miz1 to suppress cell adhesion and induce apoptosis. J. Cell Biol..

[47] Walhout A.J., Temple G.F., Brasch M.A., Hartley J.L., Lorson M.A., van den Heuvel S., Vidal M. (2000). GATEWAY recombinational cloning: application to the cloning of large numbers of open reading frames or ORFeomes. Methods Enzymol..

[48] Pellet J., Tafforeau L., Lucas-Hourani M., Navratil V., Meyniel L., Achaz G., Guironnet-Paquet A., Aublin-Gex A., Caignard G., Cassonnet P. (2010). ViralORFeome: an integrated database to generate a versatile collection of viral ORFs. Nucleic Acids Res..

[49] Fugger K., Mistrik M., Danielsen J.R., Dinant C., Falck J., Bartek J., Lukas J., Mailand N. (2009). Human Fbh1 helicase contributes to genome maintenance via pro- and anti-recombinase activities. J. Cell Biol..

[50] Hung L.Y., Chen H.L., Chang C.W., Li B.R., Tang T.K. (2004). Identification of a novel microtubule-destabilizing motif in CPAP that binds to tubulin heterodimers and inhibits microtubule assembly. Mol. Biol. Cell.

[51] Yang F., DeBeaumont R., Zhou S., Naar A.M. (2004). The activator-recruited cofactor/Mediator coactivator subunit ARC92 is a functionally important target of the VP16 transcriptional activator. Proc. Natl. Acad. Sci. U.S.A..

[52] Fricke B., Heink S., Steffen J., Kloetzel P.M., Kruger E. (2007). The proteasome maturation protein POMP facilitates major steps of 20S proteasome formation at the endoplasmic reticulum. EMBO Rep..

[53] Ricci E.P., Mure F., Gruffat H., Decimo D., Medina-Palazon C., Ohlmann T., Manet E. (2009). Translation of intronless RNAs is strongly stimulated by the Epstein-Barr virus mRNA export factor EB2. Nucleic Acids Res..

[54] Albers M., Kranz H., Kober I., Kaiser C., Klink M., Suckow J., Kern R., Koegl M. (2005). Automated yeast two-hybrid screening for nuclear receptor-interacting proteins. Mol. Cell. Proteomics.

[55] Tafforeau L., Rabourdin-Combe C., Lotteau V. (2012). Virus-human cell interactomes. Methods Mol Biol..

[56] Juillard F., Bazot Q., Mure F., Tafforeau L., Macri C., Rabourdin-Combe C., Lotteau V., Manet E., Gruffat H. (2012). Epstein-Barr virus protein EB2 stimulates cytoplasmic mRNA accumulation by counteracting the deleterious effects of SRp20 on viral mRNAs. Nucleic Acids Res..

[57] Vidalain P.O., Boxem M., Ge H., Li S., Vidal M. (2004). Increasing specificity in high-throughput yeast two-hybrid experiments. Methods.

[58] Pellet J., Meyniel L., Vidalain P.O., de Chassey B., Tafforeau L., Lotteau V., Rabourdin-Combe C., Navratil V. (2009). pISTil: a pipeline for yeast two-hybrid Interaction Sequence Tags identification and analysis. BMC Res. Notes.

[59] Young D.B., Krauer K., Kienzlz N., Sculley T. (1997). Both A type and B type Epstein-Barr virus nuclear antigen 6 interact with RBP-2N. J. Gen. Virol..

[60] Krauer K.G., Belzer D.K., Liaskou D., Buck M., Cross S., Honjo T., Sculley T. (1998). Regulation of interleukin-1b transcription by Epstein-Barr virus involves a number of latent proteins via their interaction with RBP. Virology.

[61] Cooper A., Johannsen E., Maruo S., Cahir-McFarland E., Illanes D., Davidson D., Kieff E. (2003). EBNA3A association with RBP-Jkappa down-regulates c-myc and Epstein-Barr virus-transformed lymphoblast growth. J. Virol..

[62] Calderwood M.A., Venkatesan K., Xing L., Chase M.R., Vazquez A., Holthaus A.M., Ewence A.E., Li N., Hirozane-Kishikawa T., Hill D.E. (2007). Epstein-Barr virus and virus human protein interaction maps. Proc. Natl. Acad. Sci. U.S.A..

[63] Lin J., Johannsen E., Robertson E., Kieff E. (2002). Epstein-Barr virus nuclear antigen 3C putative repression domain mediates coactivation of the LMP1 promoter with EBNA-2. J. Virol..

[64] Katsanis N., Fisher E.M. (1998). A novel C-terminal binding protein (CTBP2) is closely related to CTBP1, an adenovirus E1A-binding protein, and maps to human chromosome 21q21.3. Genomics.

[65] Johnson B.D., Schumacher R.J., Ross E.D., Toft D.O. (1998). Hop modulates Hsp70/Hsp90 interactions in protein folding. J. Biol. Chem..

[66] Kim J., Kim J.H., Lee S.H., Kim D.H., Kang H.Y., Bae S.H., Pan Z.Q., YS S. (2002). The novel human DNA helicase hFBH1 is an F-box protein. J. Biol. Chem..

[67] Si J., Yu X., Zhang Y., DeWille J.W. (2010). Myc interacts with Max and Miz1 to repress C/EBPδ promoter activity and gene expression. Mol. Cancer.

[68] Do-Umehara H.C., Chen C., Urich D., Zhou L., Qiu J., Jang S., Zander A., Baker M.A., Eilers M., Sporn P.H.S. (2013). Suppression of inflammation and acute lung injury by Miz1 via repression of C/EBP-δ. Nat. Immunol..

[69] Yenamandra S.P., Hellman U., Kempkes B., Darekar S.D., Petermann S., Sculley T., Klein G., Kashuba E. (2010). Epstein-Barr virus encoded EBNA-3 binds to vitamin D receptor and blocks activation of its target genes. Cell. Mol. Life Sci..

[70] Wanzel M., Kleine-Kohlbrecher D., Herold S., Hock A., Berns K., Park J., Hemmings B., Eilers M. (2005). Akt and 14–3–3eta regulate Miz1 to control cell-cycle arrest after DNA damage. Nat. Cell Biol..

[71] Herold S., Wanzel M., Beuger V., Frohme C., Beul D., Hillukkala T., Syvaoja J., Saluz H.-P., Haenel F., Eilers M. (2002). Negative regulation of the mammalian UV response by Myc through association with Miz-1. Mol. Cell.

[72] Allday M.J., Sinclair A., Parker G., Crawford D.H., Farrell P.J. (1995). Epstein-Barr virus efficiently immortalizes human B cells without neutralizing the function of p53. EMBO J..

[73] Allday M.J., Inman G.J., Crawford D.H., Farrell P.J. (1995). DNA damage in human B cells can induce apoptosis, proceeding from G1/S when p53 is transactivation competent and G2/M when it is transactivation defective. EMBO J..

[74] Bajaj B.G., Murakami M., Cai Q., Verma S.C., Lan K., Robertson E.S. (2008). Epstein-Barr virus nuclear antigen 3C interacts with and enhances the stability of the c-Myc oncoprotein. J. Virol..

[75] Yin X., Giap C., Lazo J.S., Prochownik E.V. (2003). Low molecular weight inhibitors of Myc-Max interaction and function. Oncogene.

[76] Jin J., Cai Y., Yao T., Gottschalk A.J., Florens L., Swanson S.K., Gutierrez J.L., Coleman M.K., Workman J.L., Mushegian A. (2005). A mammalian chromatin remodeling complex with similarities to the yeast INO80 complex. J. Biol. Chem..

[77] Douville J.M., Cheung D.Y., Herbert K.L., Moffatt T., Wigle J.T. (2011). Mechanisms of MEOX1 and MEOX2 regulation of the cyclin dependent kinase inhibitors p21 and p16 in vascular endothelial cells. PLoS One.

[78] Borggrefe T., Yue X. (2011). Interactions between subunits of the mediator complex with gene-specific transcription factors. Semin. Cell Dev. Biol..

[79] Dolfini D., Gatta R., Mantovani R. (2012). NF-Y and the transcriptional activation of CCAAT promoters. Crit. Rev. Biochem. Mol. Biol..

[80] Paschos K., Smith P., Anderton E., Middeldorp J.M., White R.E., Allday M.J. (2009). Epstein-Barr virus latency in B cells leads to epigenetic repression and CpG methylation of the tumour suppressor gene Bim. PLoS Pathog..

[81] Hughes R., Kristiansen M., Lassot I., Desagher S., Mantovani R., Ham J. (2011). NF-Y is essential for expression of the proapoptotic bim gene in sympathetic neurons. Cell Death Differ..

[82] Liu C.D., Chen Y.L., Min Y.L., Zhao B., Cheng C.P., Kang M.S., Chiu S.J., Kieff E., Peng C.W. (2012). The nuclear chaperone nucleophosmin escorts an Epstein-Barr virus nuclear antigen to establish transcriptional cascades for latent infection in human B cells. PLoS Pathog..

[83] Wiese K.E., Walz S., von Eyss B., Wolf E., Athineos D., Sansom O., Eilers M. (2013). The role of MIZ-1 in MYC-dependent tumorigenesis. Cold Spring Harb. Perspect. Med..

[84] Morandell D., Kaiser A., Herold S., Rostek U., Lechner S., Mitterberger M.C., Jansen-Dürr P., Eilers M., Zwerschke W. (2012). The human papillomavirus type 16 E7 oncoprotein targets Myc-interacting zinc-finger protein-1. Virology.

