# Supplementary material for: Epstein–Barr virus nuclear antigen 3A protein regulates CDKN2B transcription via interaction with MIZ-1
Source: Nucleic Acids Res. 2014 Aug 4;42(15):9700–16. doi: 10.1093/nar/gku697 (PMC4150796; doi:10.1093/nar/gku697)
Supplement: SUPPLEMENTARY DATA [file supp_42_15_9700__index.html]

Epstein–Barr virus nuclear antigen 3A protein regulates CDKN2B transcription via interaction with MIZ-1 — Epstein–Barr virus nuclear antigen 3A protein regulates CDKN2B transcription via interaction with MIZ-1 — SUPPLEMENTARY DATA 

# Epstein–Barr virus nuclear antigen 3A protein regulates CDKN2B transcription via interaction with MIZ-1

## SUPPLEMENTARY DATA

**Files in this Data Supplement:**

- SUPPLEMENTARY DATA
