# Supplementary material for: Epstein–Barr virus nuclear antigen 3A protein regulates CDKN2B transcription via interaction with MIZ-1
Source: Nucleic Acids Res. 2014 Aug 4;42(15):9700–16. doi: 10.1093/nar/gku697 (PMC4150796; doi:10.1093/nar/gku697)
Supplement: SUPPLEMENTARY DATA [file supp_gku697_nar-00675-x-2014-File011.pdf]

Supplementary Table 1: list of qPCR primers used for mRNA expression, ChIP and oligo pulldown.

mRNA expression

Primer name	Primer sequence
ß-actin forward (Human)	GCTGCGTGTGGCTCCCGAGGAG
ß-actin reverse (Human)	ATCTCATTGTGCTGGGTGCCAG
GAPDH forward (Human)	TCCACCACCCTGTTGCTGTAG
GAPDH reverse (Human)	ACCCACTCCTCCACCTTTGAC
CDKN2B RNA forward	CGGAGTCAACCGTTTCGG
CDKN2B RNA reverse	GGTGAGAGTGGCAGGGTCTG
CDKN1C RNA forward	ATGTCCGACGCGTCCCTCC
CDKN1C RNA reverse	CGAGTCGCTGTCCACTTCGG
CDKN1A RNA forward	GGAGACTCTCAGGGTCGAAA
CDKN1A RNA reverse	GGCGTTTGGAGTGGTAGAAATC
CEBPD RNA forward	AGCGCAACAACATCGCCGTG
CEBPD RNA reverse	GTCGGGTCTGAGGTATGGGTC

ChIP

Primer name	Primer sequence
Primer A forward	GGGCTTTGGAAGATCAAGTG
Primer A reverse	GGCCTGGGTGGTCCTAAC
Primer B forward	CCAACGTCTCCACAGTGAAA
Primer B reverse	AATGCTGGCTGCACTGCT
Primer C forward	AGTCTCTGGCGCATGCGTCCTA
Primer C reverse	GTCGCGGAGTCCTCACTG
Primer D forward	CGGCATCTCCCATACCTG
Primer D reverse	GACCTGCCAGAGAGAGCAGA
Myoglobin forward	GGAGAAAGAAGGGGAATCACA
Myoglobin reverse	GATAAATATAGCCAACGCCACA

Oligos pulldown

Primer name	Primer sequence
p15 Inr forward	GGCTGGCTCCCCACTCTGCCAGAG
p15 Inr reverse	CTCTGGCAGAGTGGGGAGCCAGCC
p15 Inr mut forward	GGCTGGCTC**AA**CA**A**T**A**TGCCAGAG
p15 Inr mut reverse	CTCTGGCATATTGTTGAGCCAGCC

Supplementary Figure legends

Supplementary Figure 1: Validation of the two-hybrid screen data by co-immunoprecipitations in human cells. Expression plasmids for Flag.EBNA3A, Flag.EBNA3B, Flag.EBNA3C and the Myc-Or GFP-tagged coding sequence for the putative partner (A) or for Myc.EBNA3A, Myc.EBNA3B, Myc.EBNA3C and the Flag-tagged coding sequence for the putative partner (B) were transfected into HEK293T cells as indicated in each Figure. In almost all cases, plasmids used express the full length putative partner at the excception of Myc-CCDC80 that expresses an N-terminal truncated protein. Cellular extracts were immunoprecipitated with an M2 anti-Flag mAb affinity gel and the immunoprecipitated complexes were analysed by western blotting using an anti-Flag polyclonal Flag antibody to detect the EBNA3 proteins or an anti-Myc polyclonal antibody to detect the cellular putative partner proteins.

Supplementary Figure 2. The POZ domain and the C-terminal domain of MIZ-1 interact with different regions of EBNA3A. (A) ^{35}S-labeled EBNA3A or EBNA3A deletion mutants depicted on the left of the Figure were incubated with purified GST (lanes 2), GST-MIZ-1 830-803 or GST-Miz POZ (lanes 3) bound to glutathione sepharose beads. The bound proteins were analysed by SDS-PAGE and visualized by autoradiography. In lane 1, the equivalent of 1/12 of the EBNA3A or EBNA3A deletion mutants-expressing rabbit reticulocyte lysate used in each assay was loaded onto the gel. (B) As in A except for the use of Flag-tagged EBNA3A and EBNA3A deletion mutants.

Supplementary Figure 3. *CDKN2B* expression in lymphoblastoid cell lines is highly dependent on the presence or absence of EBNA3A. (A) EBNA3A and MIZ-1 protein levels in LCL infected with wild type EBV (LCL) or with the recombinant EBV deleted from the EBNA3A gene, were analysed by western blotting using a specific anti-EBNA3A polyclonal antibody, a MIZ-1 specific polyclonal antibody or an anti-α-tubulin mAb as internal control as indicated. (B) Analysis of *CDKN2B* mRNA expression levels from normal LCLs (wt) or LCLs established with EBNA3A-deficient recombinant viruses, E3AmtB or E3AmtA, for two different donors, by standard real time RT-PCR.

Supplementary Figure 4. Differential expression of *CDKN1A, CDKN1C* and *CEBPD* in lymphoblastoid cell lines expressing - or not - EBNA3A. Analysis of *CDKN2B, CDKN1A and CDKN1C* mRNA expression levels from a normal LCL (wt) or an LCL established from the same donor but infected with an EBNA3A-deficient recombinant virus (D2 E3AmtB3) by either standard RT-PCR using actin mRNA as an internal control (A) or by real time RT-PCR (B). Histogram bars represent values relative to housekeeping gene *GAPDH*. Error bars represent standard deviation from four independent experiments. (C) Analysis of *CEBPD* mRNA expression level from LCL (wt) and LCL (D2 E3AmtB3) by standard RT-PCR using actin mRNA as an internal control. The use of increasing dilutions of cDNAs - indicated by the black triangles - shows that the PCR is in the quantitative range.

Supplementary Figure 5. NPM does not interact with EBNA3A. Expression plasmids for Myc-EBNA3A, Flag-MIZ-1 or Flag-NPM were transfected into HeLa cells as indicated in the Figure. Cellular extracts were immunoprecipitated with an M2 anti-Flag mAb affinity gel and the immunoprecipitated complexes were analysed by western blotting using an anti-Flag polyclonal antibody to detect MIZ-1 or NPM or an anti-Myc 9E10 monoclonal antibody to detect EBNA3A. Inputs correspond to 8% of the cell extract used for immunoprecipitation.

A

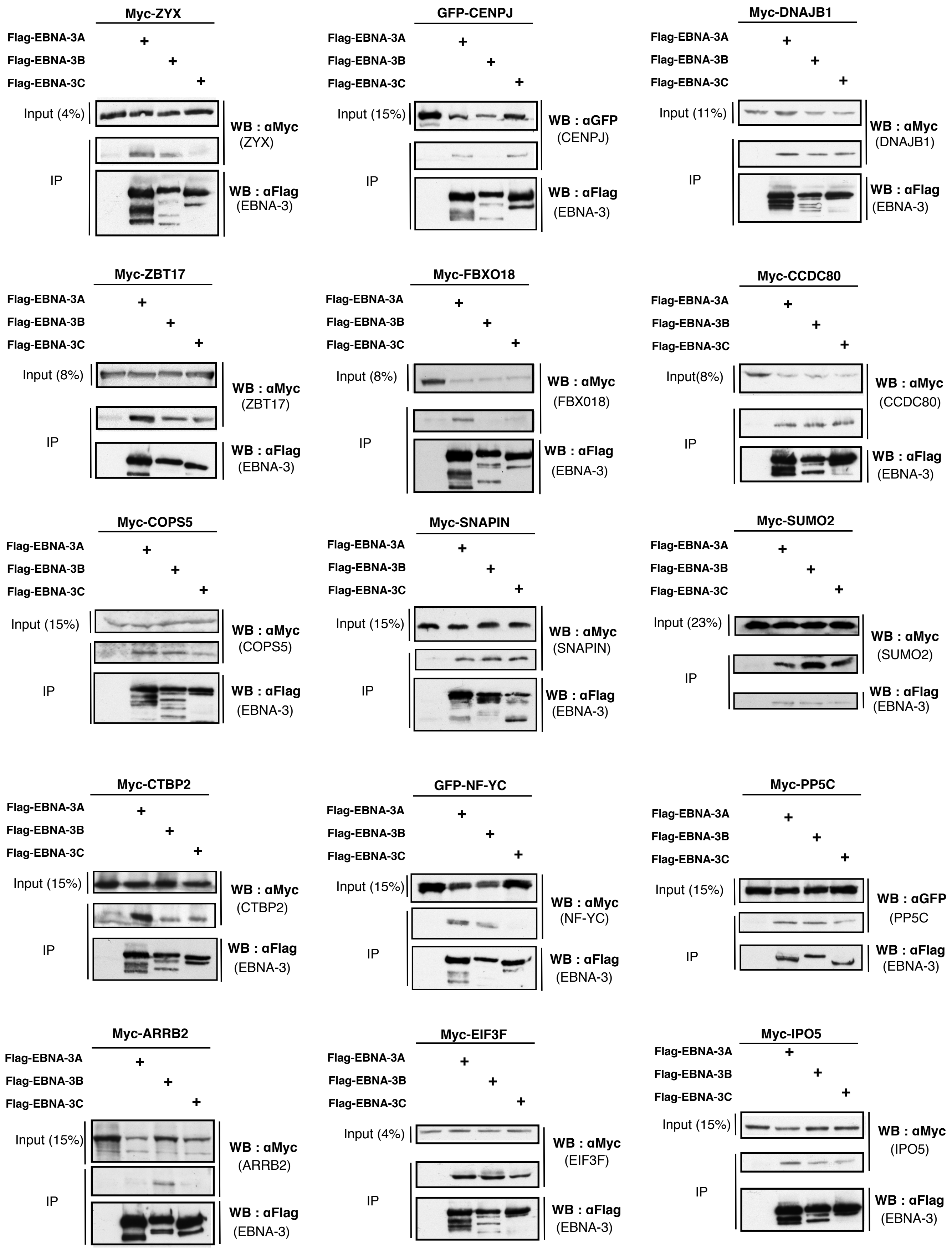
Myc-ZYX
Flag-EBNA-3A
Flag-EBNA-3B
Flag-EBNA-3C
Input (4%)
IP
WB : αMyc (ZYX)
WB : αFlag (EBNA-3)
GFP-CENPJ
Input (15%)
WB : αGFP (CENPJ)
WB : αFlag (EBNA-3)
Myc-DNAJB1
Input (11%)
WB : αMyc (DNAJB1)
WB : αFlag (EBNA-3)
Myc-ZBT17
Input (8%)
WB : αMyc (ZBT17)
WB : αFlag (EBNA-3)
Myc-FBXO18
Input (8%)
WB : αMyc (FBX018)
WB : αFlag (EBNA-3)
Myc-CCDC80
Input(8%)
WB : αMyc (CCDC80)
WB : αFlag (EBNA-3)
Myc-COPS5
Input (15%)
WB : αMyc (COPS5)
WB : αFlag (EBNA-3)
Myc-SNAPIN
Input (15%)
WB : αMyc (SNAPIN)
WB : αFlag (EBNA-3)
Myc-SUMO2
Input (23%)
WB : αMyc (SUMO2)
WB : αFlag (EBNA-3)
Myc-CTBP2
Input (15%)
WB : αMyc (CTBP2)
WB : αFlag (EBNA-3)
GFP-NF-YC
Input (15%)
WB : αMyc (NF-YC)
WB : αFlag (EBNA-3)
Myc-PP5C
Input (15%)
WB : αGFP (PP5C
WB : αFlag (EBNA-3)
Myc-ARRB2
Input (15%)
WB : αMyc (ARRB2)
WB : αFlag (EBNA-3)
Myc-EIF3F
Input (4%)
WB : αMyc (EIF3F)
WB : αFlag (EBNA-3)
Myc-IPO5
Input (15%)
WB : αMyc (IPO5)
WB : αFlag (EBNA-3)

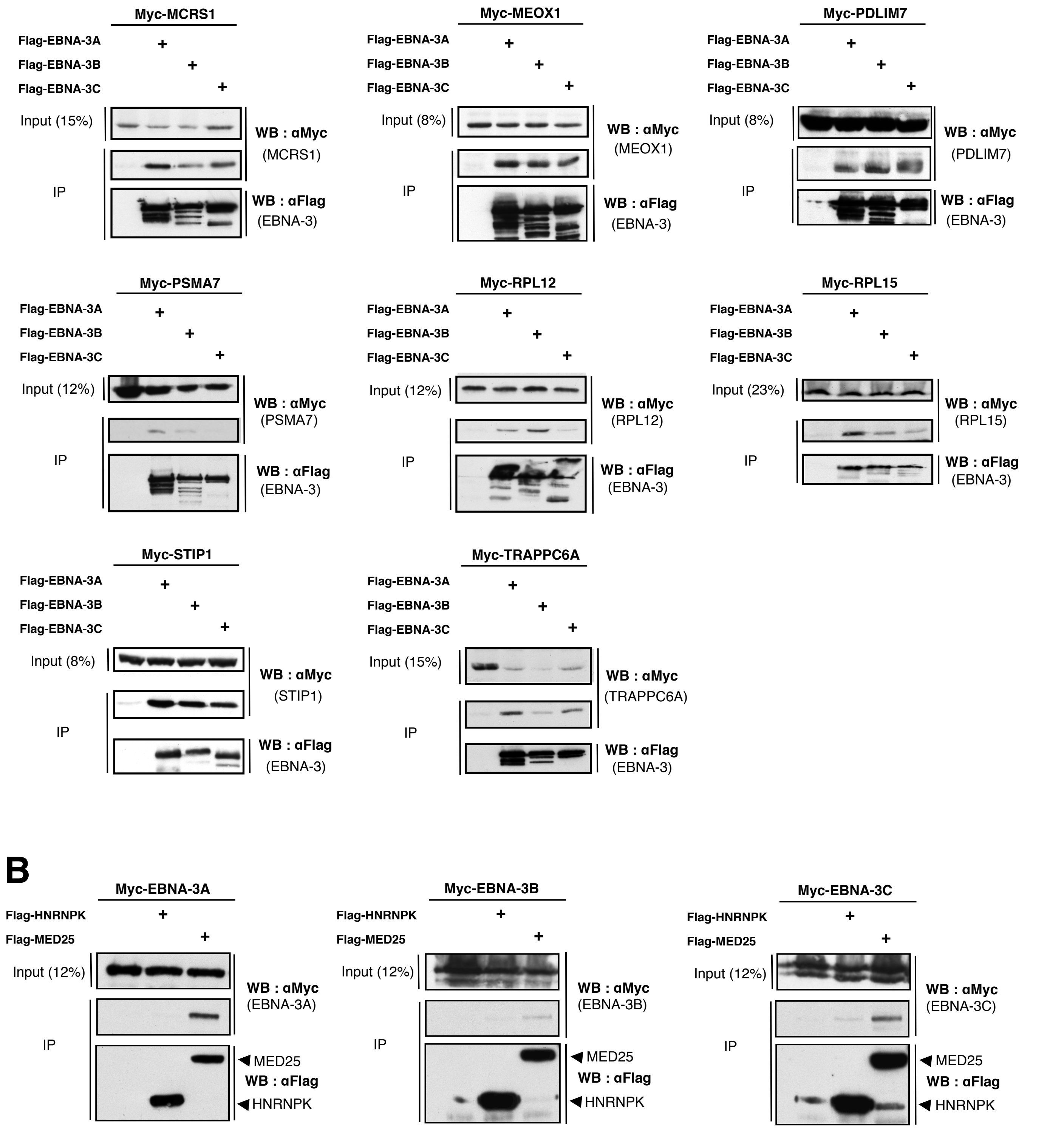

Supplementary Figure 1

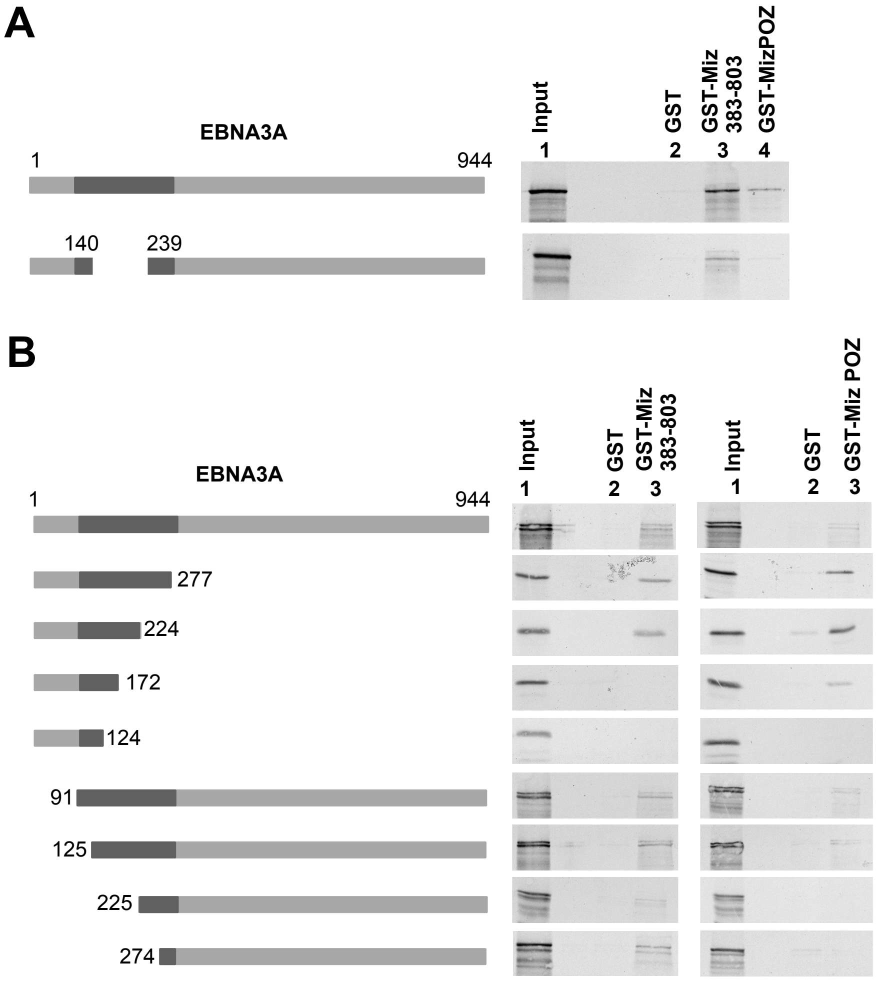

Supplementary Figure 2

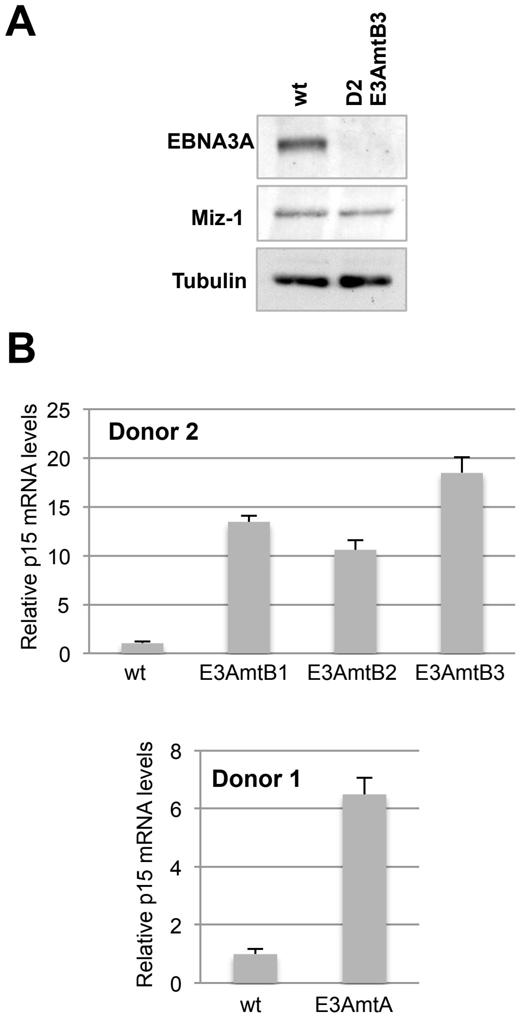

Supplementary Figure 3

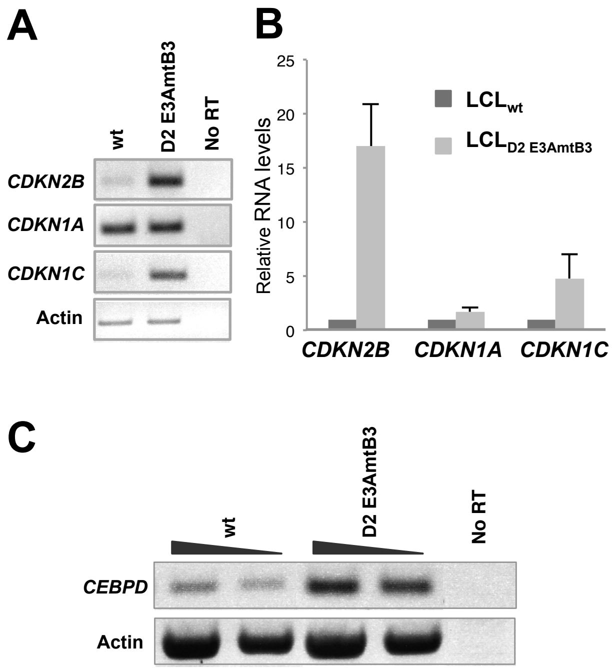

Supplementary Figure 4

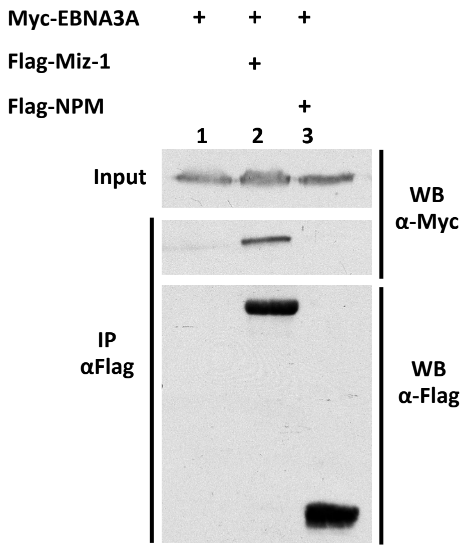

Supplementary Figure 5
